# Peptide-phosphorodiamidate morpholino oligomer therapy for dysferlinopathy induces pseudoexon skipping and restoration of functional protein

**DOI:** 10.1172/jci.insight.204742

**Published:** 2026-07-14

**Authors:** James E. Gooding, Gyeongsu Park, Atish Wagh, Jonathan K. Watts, Janice A. Dominov, Robert H. Brown

**Affiliations:** 1 Department of Neurology and; 2 RNA Therapeutics Institute, University of Massachusetts Chan Medical School, Worcester, Massachusetts, USA.

**Keywords:** Genetics, Muscle biology, Gene therapy, Genetic diseases, Skeletal muscle

## Abstract

The dysferlinopathies are a spectrum of autosomal recessive muscle diseases caused by mutations in the dysferlin gene (*DYSF*). Clinical manifestations vary from asymptomatic hyperCKemia to severe muscle pathology and loss of muscle function. These are designated as limb-girdle muscular dystrophy type 2R (LGMDR2; formerly LGMD2B or Miyoshi myopathy). Among other functions, dysferlin is crucial for plasma membrane repair and maintenance of intracellular calcium homeostasis. In previous studies, we identified 2 independent point mutations deep within introns that cause aberrant *DYSF* mRNA splicing and the inclusion of pseudoexons within transcripts that diminish protein expression. In this study, we generated and characterized a mouse model for 1 of these mutations (within *DYSF* intron 44). In these mice, a segment of human *DYSF* DNA containing the mutant intronic sequence flanked by surrounding human exon sequences replaced the normal homologous mouse DNA. These mice exhibited aberrant *Dysf* pre-mRNA splicing, pseudoexon inclusion, loss of DYSF protein expression, and muscle pathology similar to that observed in patients. Using this model, we identified antisense oligonucleotides and a peptide-phosphorodiamidate morpholino oligomer that blocks the mouse *Dysf* pre-mRNA splicing complexes from binding the mutant pre-mRNA, thereby restoring nearly normal muscle pathology and function.

## Abstract 2

Antisense oligonucelotide therapy targeting a pseudo exon for the treatment of a muscular dystrophy

## Graphical abstract

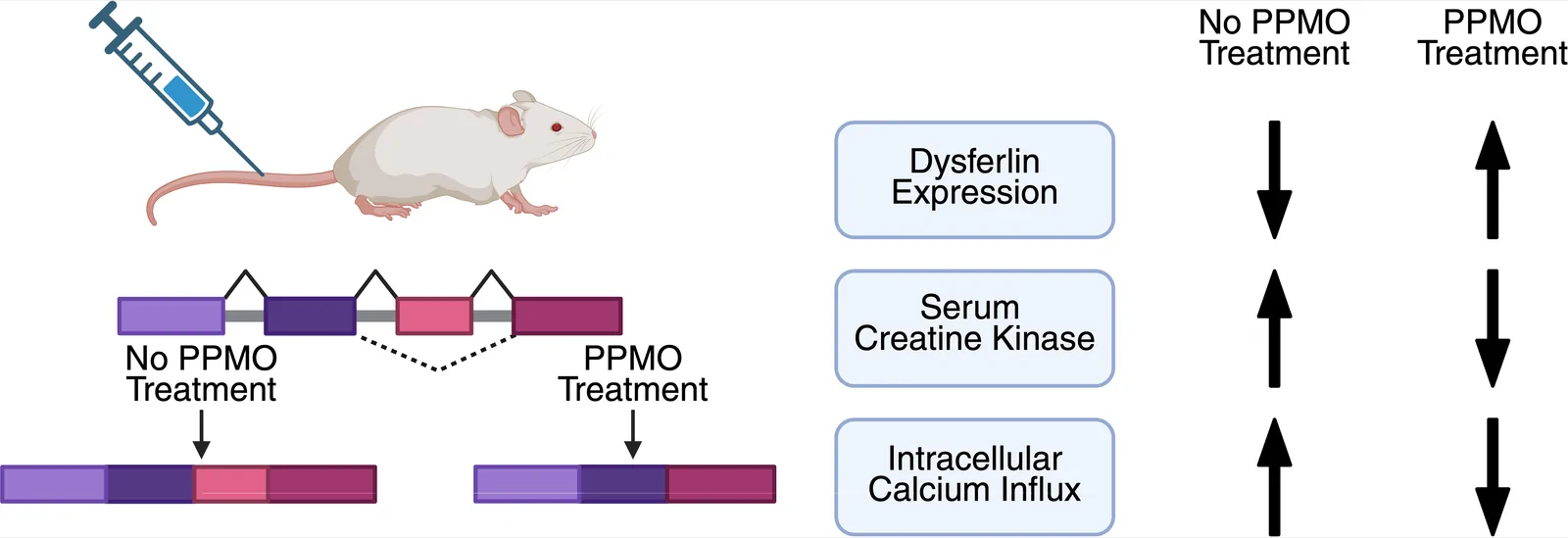

## Introduction

Dysferlinopathies are a spectrum of progressive muscular dystrophies, resulting from mutations in the dysferlin gene (1, 2). There are 2 major phenotypes: adult-onset distal muscle weakening and primary proximal muscle weakness in the pattern of a limb girdle muscular dystrophy. The distal muscle weakening phenotype particularly affects the gastrocnemius and soleus muscles, and the proximal muscle weakening phenotype particularly affects the pelvic and shoulder girdle muscles (3–6). Clinical manifestations of the dysferlinopathies can be quite variable. They typically entail adult-onset weakness in 1 of the above 2 phenotypes associated with marked serum elevations of the muscle enzyme creatine kinase (CK). However, some cases have presented with only cramping or even no manifestations other than serum CK elevations. Pathologically, the dysferlinopathies reveal muscle degeneration, replacement of muscle tissue by fat and scar tissue, and myofiber size variation, often with inflammatory phenotypes (7). As with other forms of muscular dystrophy, there is currently no primary treatment to cure dysferlinopathies.

The *DYSF* gene is located on chromosome 2p13.3-13.1, spans over 232 kb of genomic DNA, and codes for a 6.2 kb major muscle transcript encompassing 55 exons that translate into a 237 kDa protein (8). This large dysferlin protein contains several functional structural domains. There are seven C2 domains that bind Ca^2+^ and lipids with varying affinities (6, 9, 10). Dysferlin forms multiprotein complexes with proteins such as AHNAK, MG53, and TRIM72, which bind C2 domains near its N-terminus, as well as additional proteins for which precise dysferlin binding sites are not defined, including annexin A1, annexin A2, and calpain-3 (11–14). A single C-terminal transmembrane sequence anchors the protein to the membrane. These appear to serve several essential roles, including sarcolemmal membrane repair, T-tubule structure and function, vesicle trafficking, and endocytosis (6, 7, 15–18). There are 14 different isoforms of human *DYSF*, derived from the use of 2 different promoters and alternative exon splicing. These can be expressed in a variety of tissues, with isoform 8 (RefSeq accession NM_003494.4), composed of 55 exons, being predominant in skeletal muscle (19).

Pre-mRNA splicing, catalyzed by the spliceosome in the cell nucleus, removes introns from newly transcribed pre-mRNA to produce mature mRNA. Alternative splicing, through which exonic sequences are selectively included or skipped in the mRNA, allows a single gene to encode multiple protein isoforms. Alternative splicing is a highly controlled process involving several steps: splice site selection, spliceosome assembly, and the regulation of splicing elements (20, 21). Disruptions in the splicing process can lead to aberrant splicing, resulting in abnormal mature mRNA production and faulty protein generation, which may be pathogenic. Aberrant splicing events, such as the loss of an exon or the retention of intronic sequences as pseudoexons in mature mRNA, have been reported in many genes and are fundamental to the pathology of multiple disorders (22–28). Modulation of pre-mRNA splicing has therefore emerged as a powerful therapeutic strategy in many genetic conditions. By targeting pre-mRNA directly, it is possible to correct splicing defects and restore normal mRNA and protein production, offering promising potential for treating diseases caused by splicing errors (22, 29–32).

Splice-switching oligonucleotides are a major technology that can modulate alternate splicing by targeting pre-mRNA and blocking aberrant interactions between splicing factors and their pre-mRNA targets in the nucleus, thereby altering splicing outcomes (29, 33–35). To date, the FDA has approved 6 splice-switching antisense oligonucleotides (ASOs) targeting dystrophin for the treatment of Duchenne muscular dystrophy and survival motor neuron 2 for the treatment of spinal muscular atrophy (36–42). However, no ASOs have been approved for the treatment of dysferlinopathies. Indeed, there have been no therapies of any kind approved for dysferlinopathy; an intramuscular dual AAV vector approach to deliver a full-length dysferlin gene showed promise in animal models (43) and reached phase I clinical trials (ClinicalTrials.gov NCT05906251 and NCT02710500), but this approach has not advanced further.

Disruptions of splicing of the dysferlin gene can be pathogenic (44). In previous work, we identified 2 separate pathogenic mutations in patients with dysferlinopathy that lead to altered *DYSF* pre-mRNA splicing and aberrant retention of intronic sequences (pseudoexons) in the mature RNA. In both instances, the pseudoexon diminishes protein expression. These splice-altering point mutations are located deep within introns, 1 in *DYSF* intron 44 [c.4886+1249(G>T), creating pseudoexon 44.1, PE44.1] (45) and the other in *DYSF* intron 50 [c.5668-824(C>T), creating pseudoexon 50.1, PE50.1] (46). In both cases, we showed that strategies to correct this mis-splicing in patient-derived muscle cell cultures restored expression of the normal *DYSF* transcripts and DYSF protein (45, 46).

The present investigation was undertaken to translate the success of splice modulation therapy from in vitro myotubes to dysferlin mutant muscle in vivo. We first produced and characterized a mouse model of the PE44.1 mutation that carries a large segment of human DNA (*DYSF* exon 44 + mutant intron 44i + exon 45) in place of the mouse *Dysf* exon 44 + intron 44i + exon 45 sequence. Aberrant *Dysf* splicing and PE44 inclusion were recapitulated in this mouse model, resulting in typical dysferlin-deficient pathology. Using this mouse model, we next developed a potential therapy using splice-switching oligonucleotides to treat the aberrant splicing and restore normal dysferlin function in vivo.

## Results

### Generation of DYSF-PE44.1 mutant mice.

**Figure 1:**
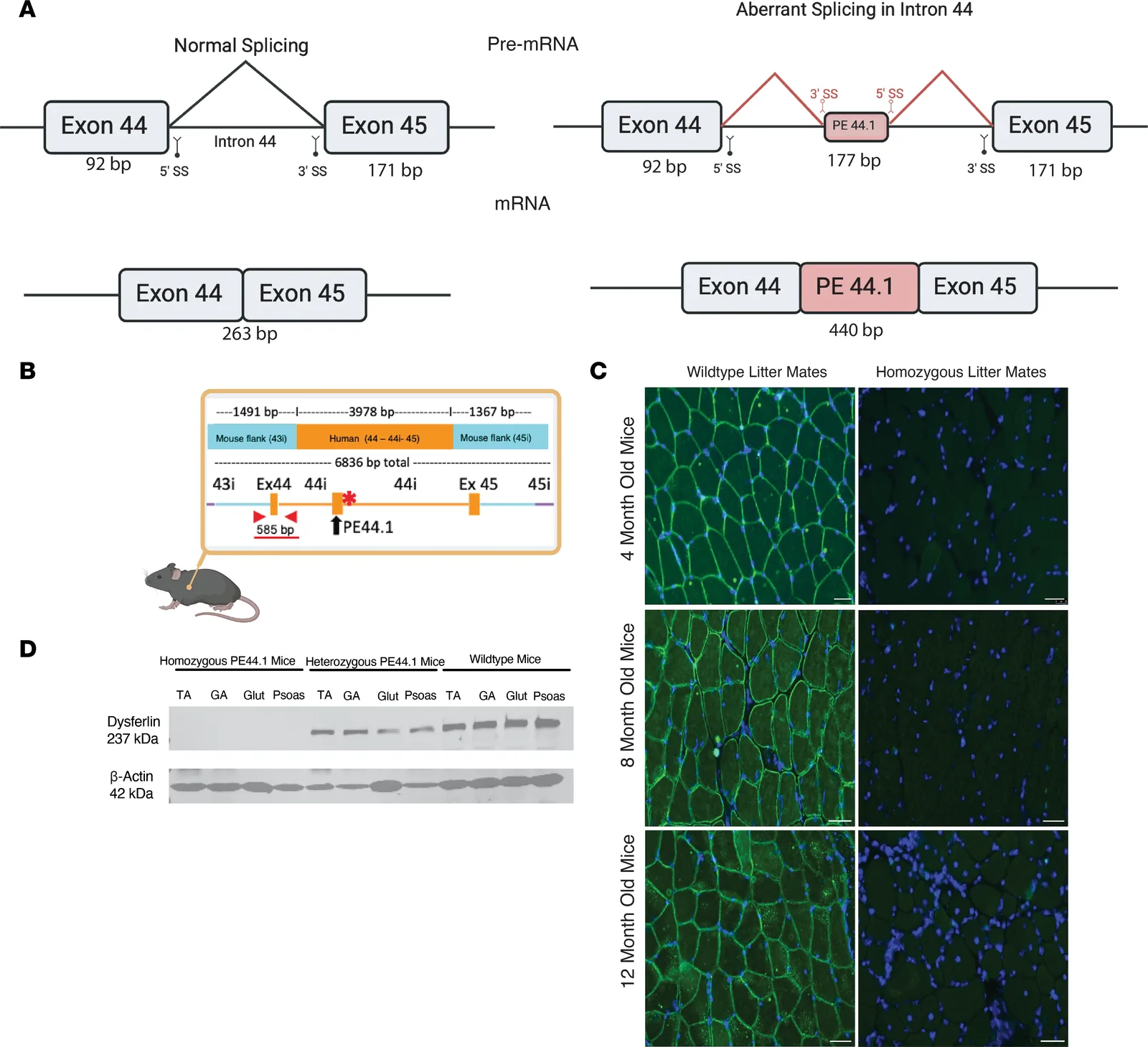
The deep intronic mutation creates a cryptic splice donor site within intron 44, resulting in a pseudoexon inclusion. (**A**) Schematic representation of normal splicing of exons 44 and 45 of WT *DYSF* mRNA and aberrant splicing caused by c.4886+1249(G>T) that generates an intronic cryptic splice site (ss) that results in the insertion of a pseudoexon into the mature *DYSF* mRNA. (**B**) Diagram of donor DNA used to generate the DYSF-PE44.1 mutant mouse with human pseudoexon mutation. Star indicates the location of the point mutation leading to PE44.1 inclusion. Arrowheads are primers used for routine genotyping. (**C**) Immunohistology of gluteus maximus muscle tissue detecting dysferlin (green) and DAPI (blue). Scale bar: 50 um. (**D**) Western blot from muscle tissue lysates detecting dysferlin and β-actin. TA, tibialis anterior; GA, gastrocnemius; Glut, gluteus maximus.

**Figure 2:**
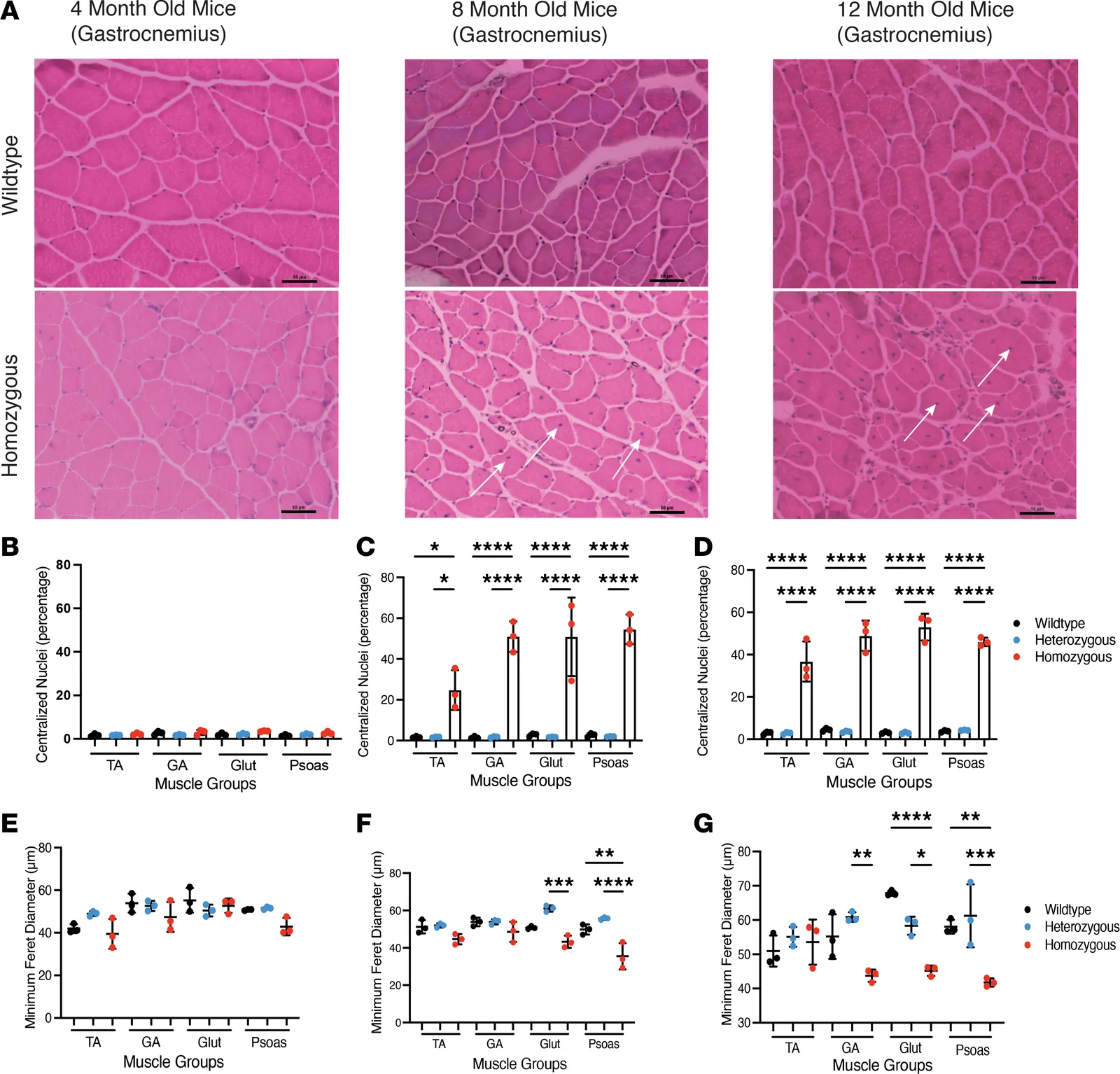
Histopathology of 4-, 8-, and 12-month-old mice. (**A**) Representative H&E staining of the gluteus maximus muscles in PE44.1 model of dysferlinopathy across age points. Scale bar: 50 um. (**B**–**D**) Quantification of fibers with centralized nuclei (arrows) in 4 muscle groups of PE44.1 homozygous, heterozygous, and WT mice at (**B**) 4 months, (**C**) 8 months, and (**D**) 12 months of age. (**E**–**G**) Quantification of minimum Feret diameter in 4 muscle groups of PE44.1 homozygous, heterozygous, and WT mice at (**E**) 4 months, (**F**) 8 months, and (**G**) 12 months of age. Statistical differences were determined using 1-way ANOVA and Tukey’s post hoc test: *P < 0.05, **P < 0.005, ***P < 0.0002, and ****P < 0.0001. TA, tibialis anterior; GA, gastrocnemius; Glut, gluteus maximus.

Previously, we identified patients with Miyoshi myopathy who lacked detectable DYSF protein in both their muscle tissue and monocytes. These patients were found to bear compound heterozygous loss-of-function mutations in DYSF; 1 allele harbored a nonsense mutation in DYSF exon 32, and the other allele contained an intronic point mutation deep within DYSF intron 44i (45). This point mutation causes aberrant splicing and expression of a pseudoexon, leading to the insertion of 177 additional nucleotides between exons 44 and 45 that maintains the reading frame and inserts 59 additional amino acids, disrupting protein stability (Figure 1A). We generated mutant mice carrying a large segment of human DNA to model this splicing mutation and provide a tool to evaluate therapeutic interventions relevant to this allele. DYSF-PE44.1 mutant mice were produced using CRISPR/Cas9-directed mutagenesis that allowed homologous recombination of a large (6.8 kb) mouse-human-mouse DNA donor sequence into the mouse Dysf locus (Figure 1B and Supplemental Figure 1A; supplemental material available online with this article; https://doi.org/10.1172/jci.insight.204742DS1). This allowed the replacement of mouse Dysf exon 44 – intron 44i – exon 45 with the homologous human exon 44 – intron 44i – exon 45 sequence, including the intron 44i c.4886+1249(G>T) mutation responsible for PE44.1 retention in mature Dysf mRNA. We generated 2 founder mice, Dysf-PE44.1 line 1 and line 2, that carried the successful germline mutation event, as confirmed by Southern blot analysis and diagnostic short- and long-range PCR (Figure 2, Supplemental Figure 1B, Supplemental Figure 2, and Supplemental Figure 3A). All subsequent experiments were performed using Dysf-PE44.1 line 1.

We confirmed that the mutant Dysf-PE44.1 mRNA was expressed in the muscles of mutant mice (Supplemental Figure 3B and Supplemental Figure 4). This result demonstrated that the mouse splicing machinery recognized the human sequence responsible for splicing human exon 44–intron 44i–exon 45 transcripts as well as the G>T point mutation leading to PE44.1 expression. Sanger sequencing of RT-PCR products derived from mutant mouse muscles, using primers spanning mouse exon 43 to exon 47, confirmed the accurate splicing of all splice junctions in this region, including the human sequences in exon 44–PE44.1–exon 45, as expected (Supplemental Figure 4). This model, therefore, recapitulates the aberrant human DYSF splicing event in the mouse context and provides a useful humanized tool to evaluate therapeutic strategies targeting the human mutation.

### Molecular characterization of Dysf-PE44.1 mouse model. Dysf-PE44.

1 heterozygous and homozygous mice exhibited normal viability. Heterozygous breeding demonstrated normal biological sex and Mendelian genotypic ratios (Supplemental Figure 5A). Additionally, body weights and grip strength for heterozygous and homozygous mice did not differ significantly from WT mice for either males or females up to the 12 months of age observed (Supplemental Figure 5, B–E).

We evaluated the expression of dysferlin expression in muscle tissues of the PE44.1 mutant mice. Strong dysferlin expression was observed in WT mice, whereas no expression was detected in the homozygous mutants (Figure 1C). These findings were confirmed by Western blot analysis of protein expression across 4 muscle groups (psoas, gluteus, gastrocnemius, anterior tibialis) (Figure 1D). Occasionally, trace amounts of DYSF protein could be detected on immunoblots, likely arising from very low levels of transcripts that did not retain PE44.1 in the mature RNA. Further, bands were noted that are routinely seen when immunoblotting lysed muscle tissue with NCL Hamlet antibody.

In addition to the absence of dysferlin in homozygous mutant mice, a marked increase in mononucleated cells was observed at 12 months of age but not at 4 months of age. This increase was driven by an expanded population of immune cells (CD45+, CD11b+, and CD8+) (showing leukocytes, myeloid cells, and activated T cells, respectively), and fibro-adipogenic precursors (FAPs; PDGFα+) (Supplemental Figure 6).

We stained muscle sections from WT, heterozygous, and homozygous mutant mice using H&E to assess muscle morphology (Figure 2). Muscle tissue from WT littermates exhibited normal morphology, but a key distinction in homozygous mutant mice was the presence of centrally nucleated fibers, indicative of muscle degeneration and an ongoing regenerative process, as highlighted in Figure 2A (arrows). Quantification of centrally located nuclei revealed that heterozygous mutant muscles appeared similar to WT, and that homozygous mutant muscle tissue began showing significant regeneration (as indicated by central nucleation) between 4 and 8 months of age (Figure 2, B and C). By 8 months, 3 muscles were more evidently affected (psoas, gluteus, gastrocnemius, 50%–60% central nucleation) than the fourth (tibialis, 20%–30%). At 12 months, the psoas, gluteus, and gastrocnemius showed little further change, while there was an apparent increase in central nucleation in the tibialis muscle (Figure 2D). These findings suggest that the tibialis muscle is less affected in the disease process relative to the other muscles examined, consistent with earlier studies of dysferlin-deficient mice (47).

We also appraised differences in fiber size as a parameter in disease progression. At 4 months of age, there were no significant differences in muscle fiber size (as indicated by the minimum Feret diameter) between WT, heterozygous, and homozygous mutant mice, even in the most affected muscle groups (Figure 2E). However, by 8 months, significantly smaller muscle fibers were observed in the more affected mutant muscle groups, such as the psoas and gluteus, in homozygous mice, and the diameters of the gastrocnemius and tibialis were indistinguishable from controls (Figure 2F). By 12 months, the gastrocnemius in mutant mice showed a significant reduction in fiber size compared with WT mice (Figure 2G). Strikingly, despite the marked changes in the other muscles, the tibialis anterior revealed no change in diameter at 12 months. Overall, homozygous mutant mice carrying the dysferlin pseudoexon mutation exhibited signs of muscle degeneration, characterized primarily by central nucleation of muscle fibers and a decrease in muscle fiber size.

### Functional analysis in DYSF-PE44.1 mouse model.

**Figure 3:**
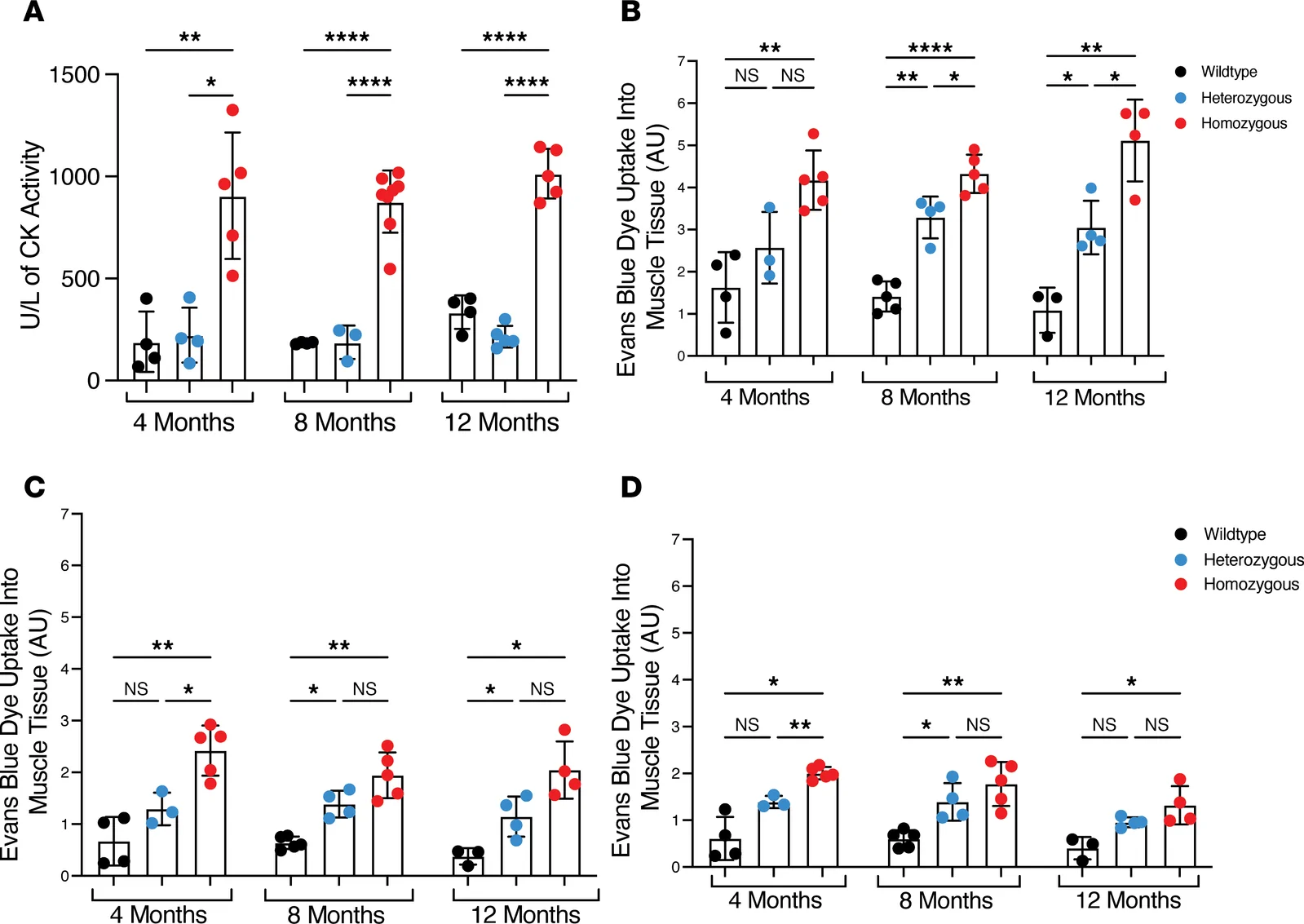
Functional assays of membrane damage due to dysferlinopathy in vivo. (**A**) Creatine kinase levels detected in the serum at 4, 8, and 12 months in WT, heterozygous, and homozygous littermates. (**B**–**D**) Evans blue dye uptake in WT, heterozygous, and homozygous littermates at 4, 8, and 12 months of age in the (**B**) psoas muscle, (**C**) gluteus muscle, and (**D**) gastrocnemius muscle. Statistical differences were determined using 1-way ANOVA and Tukey’s post hoc test: *P < 0.05, **P < 0.005, and ****P < 0.0001.

Serum CK levels are commonly used as a biomarker of muscle fiber damage caused by mechanical stress (48–50). When muscle fibers undergo mechanical stress, microtears in the sarcolemma release intracellular contents into the blood (50). In our Dysf mutant mouse model, homozygous mice carrying the pseudoexon mutation showed significantly elevated serum CK levels at 4, 8, and 12 months of age, whereas WT and heterozygous mice did not (Figure 3A).

Loss of muscle membrane integrity is also evident in damaged or diseased muscles by their increase in Evans blue dye uptake after systemic dye delivery (51). When homozygous mice carrying the pseudoexon 44.1 Dysf mutation were administered Evans blue dye, elevated levels of the dye were detected in muscle tissues, including the psoas (Figure 3B), gluteus (Figure 3C), and gastrocnemius (Figure 3D). Heterozygous mice also showed some increase in Evans blue dye uptake, although the levels were lower than those observed in homozygous mice (Figure 3C).

### ASOs induce pseudoexon skipping and restore normal DYSF mRNA and protein expression in myotubes.

We next explored approaches to correcting the mis-splicing of the mutant dysferlin pre-mRNA. We used the online tools ESE Finder 3.0 and eSkip-Finder to identify splicing regulatory elements within the pseudoexon 44.1 sequence (52, 53). The analysis revealed serine/arginine-rich protein binding sites throughout the sequence, with notable enrichment at both the beginning and near the end of the pseudoexon, showing peaks in the predicted exon skipping efficacy (Supplemental Figure 7A). For the initial screen, we designed 46 20-mer 2′-O-methoxyethyl–modified ASOs targeting the pseudoexon to promote splice exclusion (Supplemental Figure 7B and Supplemental Table 4); our previous screen included only 3 such ASOs (45). To evaluate whether the 46 candidate ASOs could induce pseudoexon skipping in *DYSF* pre-mRNA, we used myogenic cells derived from skin-primary fibroblasts from patients heterozygous for the PE44.1 pseudoexon mutation (also heterozygous for an exon 32 nonsense mutation), as previously described (45). Several ASOs reduced levels of mis-spliced transcripts as indicated by agarose gel electrophoresis (Supplemental Figure 8A). In particular, ASOs 33–37 spanned sequences that, when targeted, led to reduced pseudoexon inclusion and increased WT RNA expression. RT-qPCR and Western blotting further validated the reduction in mis-spliced transcripts, restoration in normal transcript levels, and restoration in DYSF protein (Supplemental Figure 8, B and C).

### PMOs induce pseudoexon skipping, restoring normal DYSF mRNA and protein expression in a dose-dependent manner in vitro.

**Figure 4:**
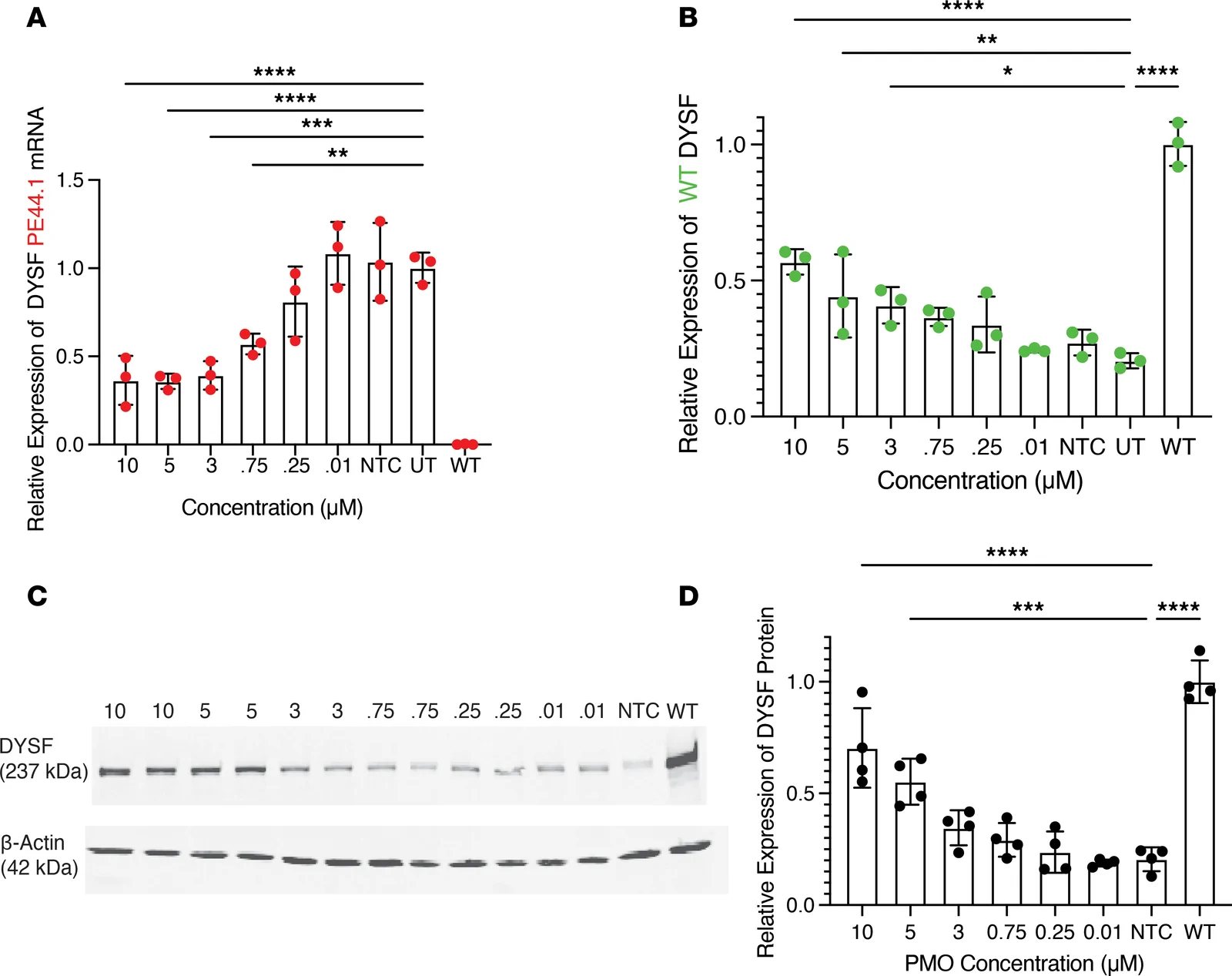
Dysferlin pseudoexon 44.1 removal via PMOs in patient-derived myotubes. (**A**–**C**) Patient-derived myotubes heterozygous for the pseudoexon 44.1 disease-causing mutation treated for 72 hours with therapeutic PMO at 10, 5, 3, 0.75, 0.25, 0.01 μM, and 1 μM of nontargeting control (NTC) PMO compared with untreated controls (UT) and untreated WT myotubes analyzed by RT-qPCR. (**A**) RT-qPCR utilizing primers amplifying pseudoexon containing dysferlin transcripts. (**B**) RT-qPCR utilizing primers amplifying WT dysferlin transcripts. (**C**) Western blot detecting dysferlin and β-actin, 2 samples per condition shown. (**D**) Quantification of dysferlin expression levels determined using the ratio to β-actin protein for normalization. Statistical differences were determined using 1-way ANOVA and Tukey’s post hoc test: **P < 0.005, ***P < 0.0002, and ****P < 0.0001.

It is established that phosphorodiamidate morpholino oligomers (PMOs) potentially provide a nontoxic, long-duration reagent for splice modulation (29, 54). Using our initial ASO data, we designed for in vivo splice modulation a 25mer PMO (designated PMO^PE44.1^) that, like the best ASOs above, targeted the region at the 3′ end of the pseudoexon (Supplemental Figure 8D and Supplemental Table 4) (55, 56). When patient-derived myotubes heterozygous for the PE44.1 intronic mutation were transfected with PMO^PE44.1^ using Gene Tools’ EndoPorter delivery reagent, we observed dose-dependent reduction in levels of mis-spliced transcripts (Figure 4A). At 10 mM PMO^PE44.1^, mis-spliced transcripts were at 30% of the untreated myotubes. Reciprocally, we observed a dose-depended restoration of the WT dysferlin transcripts, with peak levels at 5 and 10 mM PMO^PE44.1^ (Figure 4B). At the highest doses, PMO^PE44.1^ restored total dysferlin RNA expression to about 50% of the level in control myotubes, consistent with fact that only the PE44.1 allele is affected by the PMO skipping and not the exon 32 nonsense allele mutation in these cells. These findings were reflected in dose-dependent improvement in DYSF protein expression; high doses significantly restored dysferlin expression as compared with the control (Figure 4, C and D).

### Enhanced permeation of pseudoexon skipping PMO in vivo using DNA-RNA heteroduplexes or membrane-permeating peptides.

Our therapeutic goal is to develop systemic delivery of our exon-skipping PMO with broad distribution to muscle. We used 2 well-documented methods to enhance systemic uptake of PMO: use of DNA-RNA heteroduplexes (HDOs) and conjugation to cell-permeative peptides. HDOs are DNA-RNA hybrids with cholesterol conjugated to a nucleic acid strand that is complementary to PMO^PE44.1^. The cholesterol conjugate supports cellular uptake and once inside cells, the RNA in the complementary strand is vulnerable to exonuclease degradation that results in the release of the PMO (57–59). The permeating peptide chosen for this study is an iteration from the Pip series, pip9b2, modified by the addition of 4 terminal amino acids (Pip9b2^mod^) (60, 61).

We compared the efficacy of PMO^PE44.1^ alone to that of PMO^PE44.1^ modified with either HDO or the cell-permeating peptide Pip9b2^mod^ (Supplemental Figure 9). For each treatment, male and female homozygous PE44.1 mice 3 months of age received a single dose targeting the 3′ end of the pseudoexon and were evaluated 1 week after treatment (Supplemental Figure 8D). Mice treated with either the unmodified PMO^PE44.1^ or HDO-PMO^PE44.1^ were each administered 80 mg/kg or treated with Pip9b2^mod^-conjugated PMO^PE44.1^ (PPMO^PE44.1^) at a lower dose of 12.5 mg/kg. The dose was calculated from the molecular weight of the PMO alone, and the peptide was chosen as it was the most effective peptide developed for cell penetrance in the treatment of muscular dystrophies (61).

**Figure 5:**
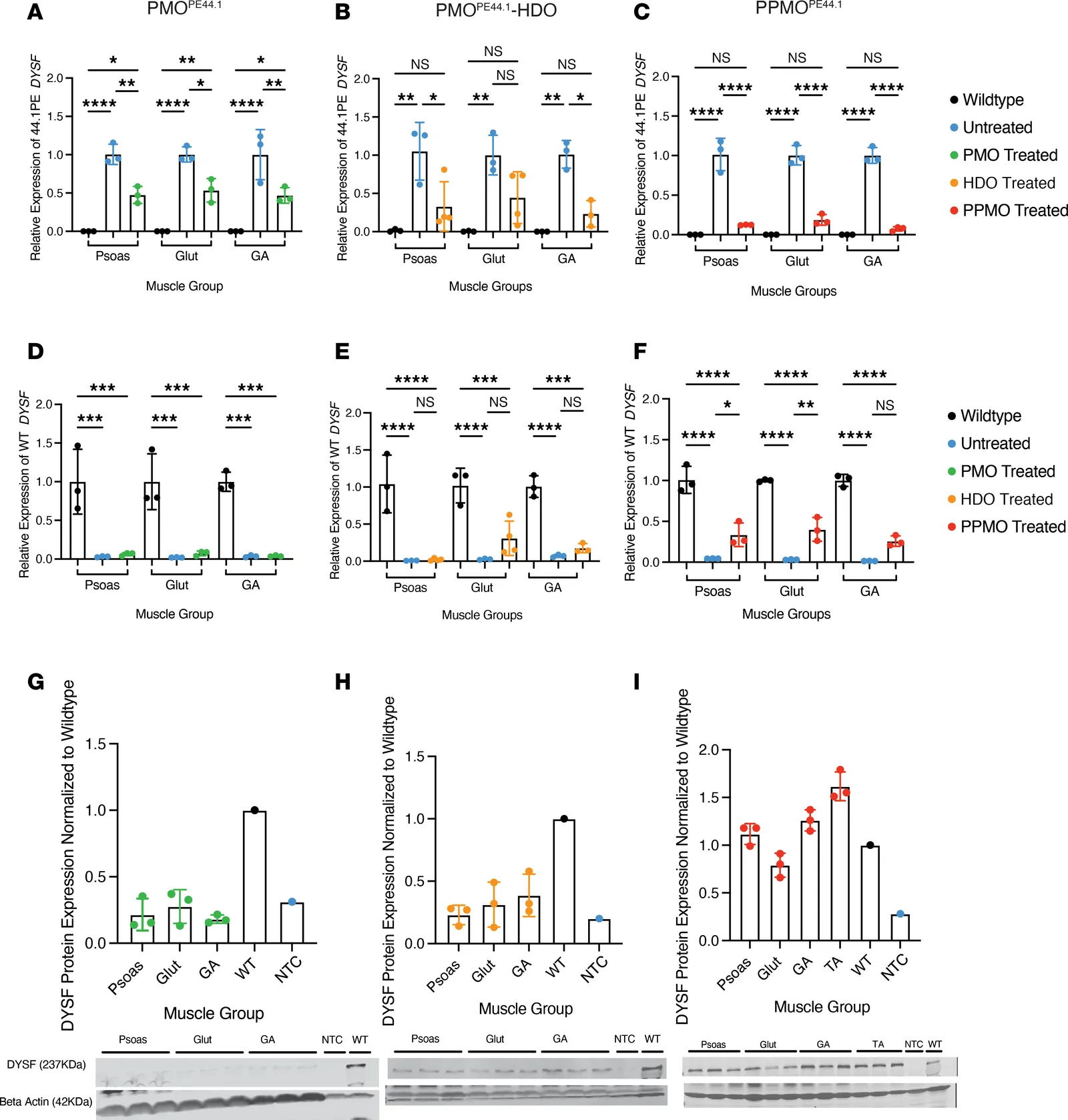
Homozygous PE44.1 model for dysferlinopathy treated with PMO and PMO variants. (**A**–**C**) mRNA expression of pseudoexon 44.1 containing transcripts evaluated using RT-qPCR from muscle tissue taken from mice treated for 1 week with (**A**) PMOPE44.1 (80 mg/kg), (**B**) PMOPE44.1-HDO (80 mg/kg), and (**C**) PPMOPE44.1 (12.5 mg/kg). (**D**–**F**) mRNA expression of WT dysferlin transcripts evaluated using RT-qPCR from muscle tissue taken from mice treated with (**D**) PMOPE44.1, (**E**) PMOPE44.1-HDO, and (**F**) PPMOPE44.1. (**G**–**I**) Western blot of muscle tissue taken from mice treated with (**G**) PMOPE44.1, (**H**) PMOPE44.1 HDO, and (**I**) PPMOPE44.1. (**A**–**F**) Legend: black, WT; blue, PBS-treated HOM mice; green, PMOPE44.1-treated HOM mice; orange, PMOPE44.1-HDO–treated HOM mice; red, PPMOPE44.1-treated HOM mice. (**A** and **D**) PMO-treated HOM mice, (**B** and **E**) HDO-treated HOM mice, (**C** and **F**) PPMO-treated HOM mice. Statistical differences were determined using 1-way ANOVA and Tukey’s post hoc test: *P < 0.05, **P < 0.005, ***P < 0.0002, and ****P < 0.0001.

When homozygous mutant mice carrying the pseudoexon 44.1 mutation were treated with PMO^PE44.1^ alone, we observed a slight reduction in mis-spliced transcripts (Figure 5A) and a minimal restoration of correctly spliced transcripts (Figure 5D), resulting in no significant improvement in protein expression (Figure 5G). In contrast, treatment with the PMO^PE44.1^-HDO more robustly decreased mis-spliced transcripts (Figure 5B) while also rescuing correctly spliced transcripts (Figure 5E). Although protein expression was improved, it was not substantially higher compared with the PMO treatment alone (Figure 5H).

The most significant improvement occurred with the PPMO^PE44.1^, which resulted in a strong reduction in mis-spliced transcripts (Figure 5C) while substantially restoring correctly spliced transcripts (Figure 5F). This restoration led to a robust increase in protein expression (Figure 5I). The elevated levels of DYSF protein were also seen in immunostaining of muscle tissue from mice treated with the PPMO^PE44.1^ (Supplemental Figure 10, A and D).

### PPMOs restored DYSF protein function in vivo.

**Figure 6:**
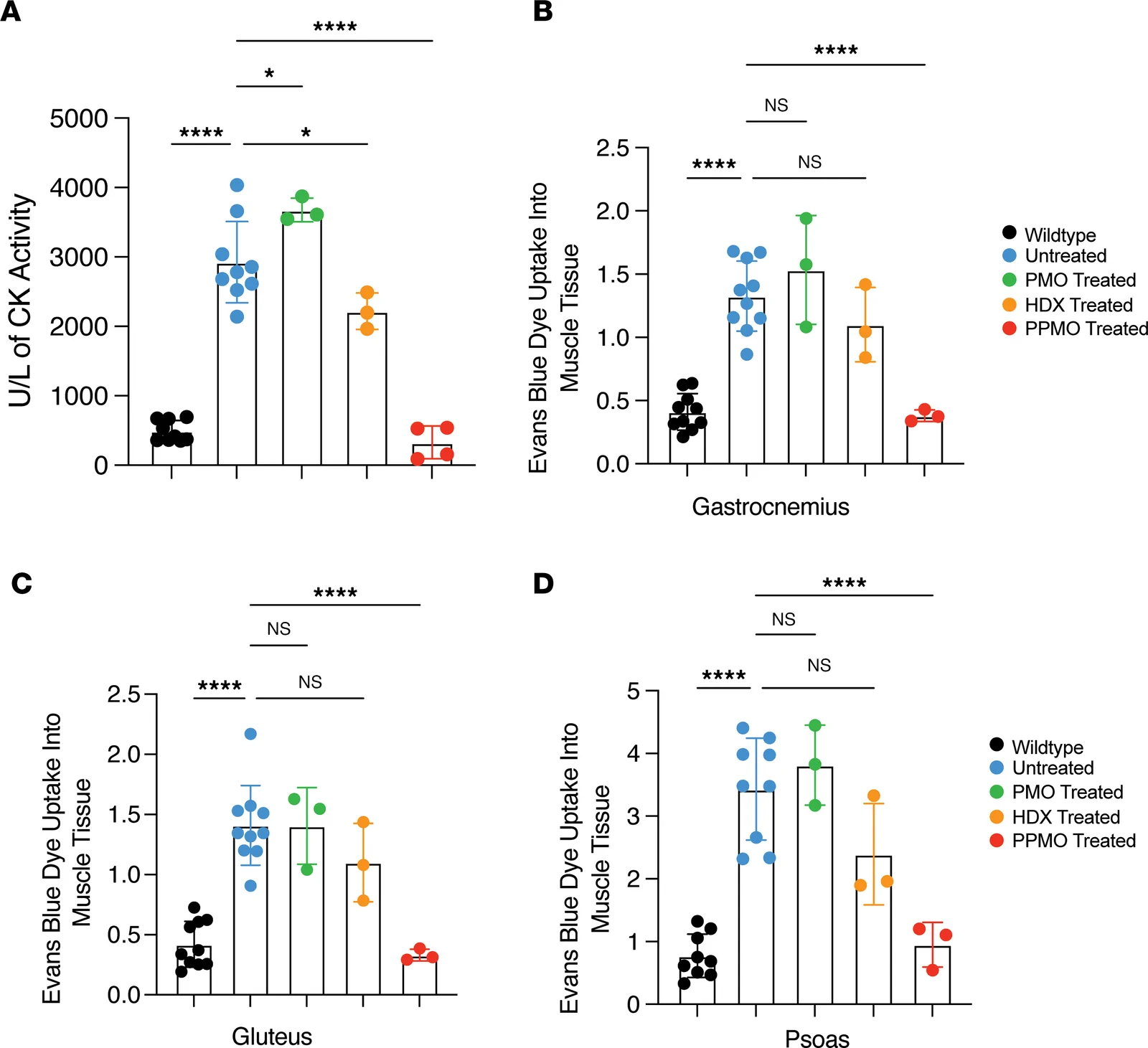
Determining functional membrane repair. (**A**) Creatine kinase levels detected in serum from mice treated with PMOPE44.1, PMOPE44.1-HDO, and PPMOPE44.1 (as in Figure 5). Evans blue dye absorption due to membrane permeability in the (**B**) gastrocnemius, (**C**) gluteus, and (**D**) psoas in mice treated with PMOPE44.1, PMOPE44.1-HDO, and PPMOPE44.1. (**A**–**D**) Legend: black, WT; blue, PBS-treated HOM mice; green, PMOPE44.1-treated HOM mice; orange, PMOPE44.1-HDO–treated HOM mice; red, PPMOPE44.1-treated HOM mice. Statistical differences were determined using 1-way ANOVA and Tukey’s post hoc test: *P < 0.05, **P < 0.005, ***P < 0.0002, and ****P < 0.0001.

Serum CK levels remained unchanged after treatment of homozygous PE44.1 mice with PMO^PE44.1^ or PMO^PE44.1^-HDO. However, treatment with PPMO^PE44.1^ restored CK activity to WT levels (Figure 6A). Similarly, the elevated levels of Evans blue dye in muscle tissue were unaffected by PMO^PE44.1^ treatment or PMO^PE44.1^-HDO, but PPMO^PE44.1^ significantly reduced Evans blue uptake (Figure 6, B–D).

**Figure 7:**
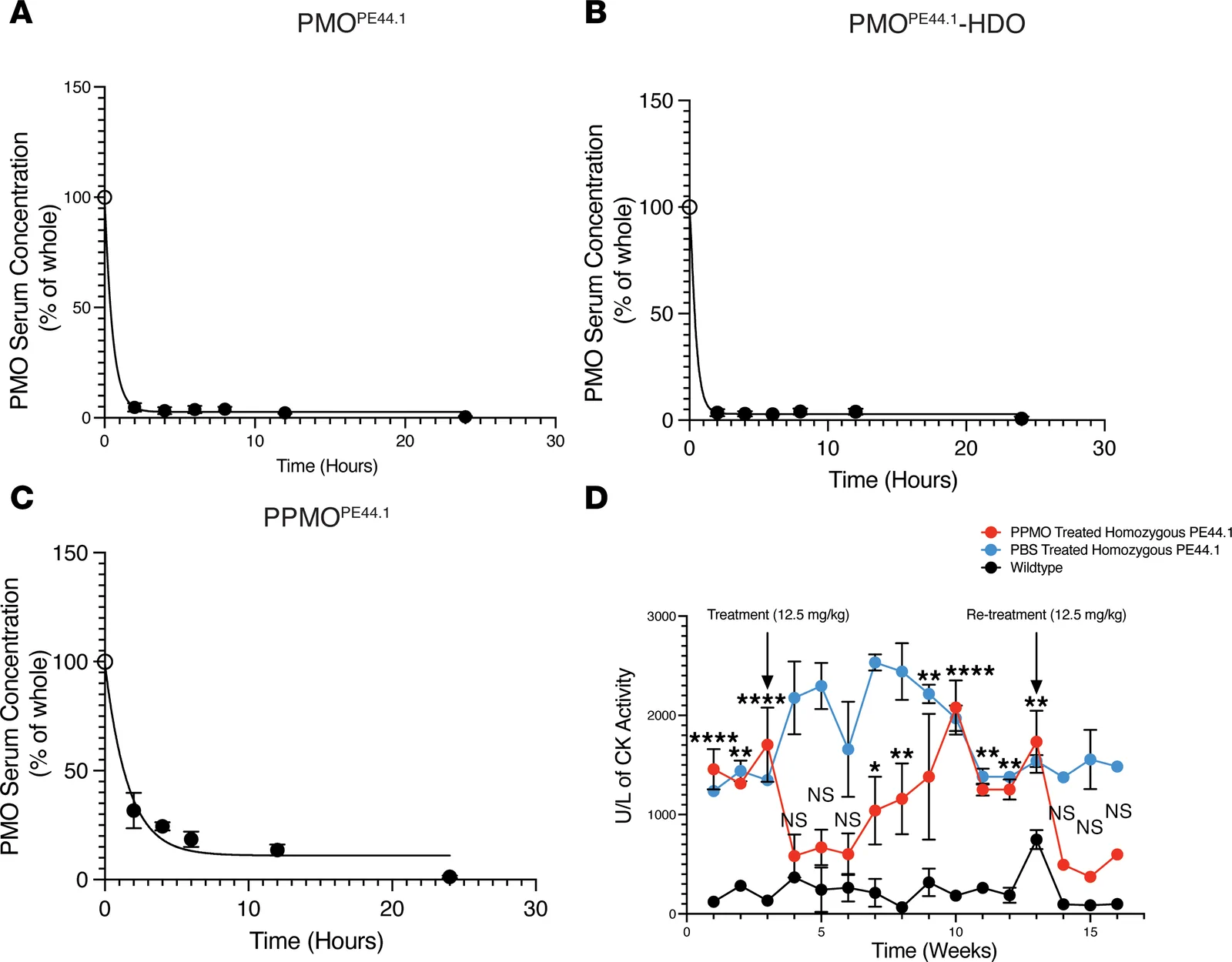
Bioavailability and functionality of PPMOPE44.1 over time. (**A**–**C**) Half-life of PMOPE44.1 in the bloodstream when delivered as (**A**) naked PMOPE44.1, (**B**) PMOPE44.1 HDO, or (**C**) PPMOPE44.1 determined via ELISA (same concentrations as Figure 5). (**D**) Creatine kinase levels detected in serum for 10 weeks after PPMOPE44.1 treatment and 3 weeks after a second PPMO treatment. One-way ANOVA and Tukey’s post hoc test: *P < 0.05, **P < 0.005, and ****P < 0.0001 comparing WT and PPMO-treated homozygous littermates.

One determinant of the effects of PPMO^PE44.1^ is the compound’s biological half-life in the blood. Using a plate-based oligonucleotide electrochemiluminescent immunoassay to quantitate PMO concentration in serum after i.v. delivery, the half-life of PMO^PE44.1^ was found to be 20 minutes (Figure 7A). By contrast, the PPMO^PE44.1^-HDO half-life was slightly increased to 1 hour (Figure 7B), and the PPMO^PE44.1^ half-life was 4 hours (Figure 7C). To explore the pharmacodynamic properties of PPMO^PE44.1^, we measured CK levels in serum longitudinally, before and after an initial PPMO^PE44.1^ treatment at 3 months of age, measuring CK levels weekly for 10 weeks, and then for 3 weeks after a second treatment at 5.5 months of age (Figure 7D). Baseline serum CK levels of 3-month-old homozygous PE44.1 mice were significantly elevated compared with WT animals before PPMO^PE44.1^ treatment. One week after the initial treatment, CK levels fell and were statistically indistinguishable from those of WT mice. The levels remained low for 3 more weeks but subsequently began to rise over the next 8–9 weeks, eventually stabilizing at elevated levels similar to baseline for 3 weeks. At that point, a second PPMO^PE44.1^ treatment was administered, and CK levels again fell to levels statistically similar to WT mice for another 3 weeks.

Tissue analyses showed accumulation of PPMO^PE44.1^ over several weeks; this occurred slowly in muscle tissue but was rapid in the kidney (Supplemental Figure 11, A and D). As an initial screen for toxicity of PPMO^PE44.1^, we dosed WT mice with PPMO^PE44.1^ (0, 4.16, 12.5, and 37.5 mg/kg) and analyzed serum electrolytes and metabolic parameters after 48 hours. The key observation was disturbance of renal function at the higher dose, as denoted by elevations in levels of blood urea nitrogen, magnesium, and KIM-1. Unlike BUN and magnesium, at the 12.5 mg/kg dose, KIM-1 levels trended toward elevation but were not statistically significant (Supplemental Figure 12). Lastly, we saw some immune reactivity in muscle tissue after 1 week of PPMO^PE44.1^ treatment, as shown in elevated levels of cells positive for CD45 (leukocyte marker) and F4/80 (macrophage marker) after PPMO^PE44.1^ treatment (Supplemental Figure 10).

## Discussion

In this study, we investigated antisense-based therapeutic strategies to correct aberrant splicing caused by a deep intronic mutation in the *DYSF* gene identified in patients with dysferlinopathy. To enable in vivo evaluation of splice-modifying therapeutics, we generated a humanized mouse model harboring the pathogenic intronic mutation (*DYSF* NM_003494.4, c.4886+1249G>T), which results in aberrant inclusion of a 177-nucleotide pseudoexon (PE44.1). Replacement of the endogenous mouse exon 44–intron 44–exon 45 region with the homologous human sequence containing the patient mutation allowed faithful recapitulation of the human splicing defect in vivo. In this context, the mouse splicing machinery recognized the cryptic splice sites within intron 44, leading to pseudoexon inclusion, loss of dysferlin protein expression, and muscle pathology that closely parallels human dysferlinopathy and established *DYSF*-null mouse models (47, 62).

Importantly, skipping of PE44.1 restores the normal *DYSF* open reading frame without introducing truncations or modified protein sequences, making this mutation particularly well-suited for splice-correction approaches. Systematic screening of ASOs targeting PE44.1 revealed that sequences directed toward the 3′ region of the pseudoexon were most effective in promoting exon skipping in patient-derived myotubes. Correspondingly, PMO and PPMO derivatives targeting this region corrected aberrant splicing, restored dysferlin protein expression both in vitro and in vivo, and led to measurable improvements in membrane repair function.

Delivery of antisense therapeutics to skeletal muscle at efficacious yet well-tolerated doses remains a major challenge for clinical translation. Although chemically modified ASOs have shown promise in neuromuscular disorders, dose-limiting toxicities, including those associated with 2′-O-methoxyethyl ASOs, have constrained their therapeutic window (63). PMOs offer an improved safety profile, but their cellular uptake is limited (55, 64). To address this, we evaluated HDO PMO (57–59, 65) formulations and peptide-conjugated PPMOs (60, 66, 67) designed to enhance tissue delivery. Consistent with prior reports, HDO PMOs improved splice correction relative to unmodified PMOs, and PPMOs achieved the most robust restoration of normal *Dysf* mRNA and protein expression. For both the PMO^PE44.1^ and PMO^PE44.1^-HDO, although some cryptic exon removal is observed, this does not result in a uniformly proportional increase in correctly spliced transcripts or protein levels. Recent reports have emphasized this phenomenon (68–70). Nevertheless, these approaches retain therapeutic potential, as even low levels of dysferlin protein expression have been shown to be meaningful (71, 72).

Notably, PPMO treatment resulted in rapid normalization of dysferlin protein levels and significant functional improvement, as evidenced by reduced serum CK levels and decreased Evans blue dye uptake, with effects persisting for several weeks after a single dose. The persistence of therapeutic effect over several weeks supports a monthly dosing regimen. This would align with dosing schedules established in current clinical trials (e.g., Pepgen, Entrada, and Sarepta) utilizing similar peptide-conjugated ASO therapies (73–75).

An initial safety assessment of the optimized PPMO demonstrated no detectable renal or hepatic toxicity at the therapeutically effective dose used in this study, although higher doses were associated with biochemical evidence of toxicity. These findings underscore the importance of continued optimization and long-term evaluation of PPMO formulations. Of particular concern is the uncertain trajectory of renal recovery after toxic insult, as well as whether repeated dosing may produce cumulative toxicity, a consideration of direct clinical relevance, as effective long-term treatment will require sustained, repeated administration of the therapeutically effective dose. Toxicity concerns center primarily on renal impact, a consideration brought into sharp focus by recent clinical setbacks involving arginine-rich PPMOs that underscore the critical need to balance delivery efficiency with safety. Encouragingly, ongoing efforts to refine peptide composition and reduce arginine content are showing promise in improving the tolerability profile of this therapeutic class of drug (76).

Although the PE44.1-causing mutation represents a rare form of dysferlinopathy, deep intronic mutations that generate pseudoexons are increasingly recognized as pathogenic mechanisms across a range of genetic diseases. The strategy described here differs fundamentally from exon-skipping approaches used in Duchenne muscular dystrophy, which restore partial protein function by generating internally truncated proteins (36, 38, 39, 41). Instead, pseudoexon skipping restores the normal full-length dysferlin transcript, analogous to the personalized ASO therapy milasen developed for a patient with Batten disease (77). The humanized PE44.1 mouse model therefore provides a valuable platform for proof-of-concept studies and for the development of therapeutic strategies applicable to other pseudoexon-driven disorders.

Together, these findings demonstrate that PPMO-mediated pseudoexon skipping can restore dysferlin expression to functionally meaningful levels in vivo and support the broader potential of antisense-based therapies for genetic diseases caused by deep intronic mutations. Although further studies will be required to define long-term efficacy, safety, and translational feasibility, this work establishes a mechanistic and preclinical foundation for targeting pathogenic pseudoexons in dysferlinopathy, and provides justification for an “*n* of 1” clinical trial approach. We have 1 remaining case in our index family and are aware of 1 other family with the same mutation. Furthermore, approximately 17% of patients with dysferlinopathy have only 1 disease-causing mutation identified by standard exon sequencing, suggesting the second allele harbors variants outside coding regions. Since there are approximately 3,000 cases of dysferlinopathy in the United States, this implies that approximately 500 current cases may be amenable to exon skipping strategies. Moreover, we note additionally that exon skipping is implicated in the pathogenesis of many genetic disorders (78).

## Methods

### Sex as a biological variable.

Both male and female sexes were included in this study. Given that no significant differences were observed between sexes, sex was not considered as a biological variable in analyses.

### Mice.

All mouse experiments were done following the regulations and guidelines set forth by the University of Massachusetts Chan Medical School IACUC. PE44.1 mutant mice, produced in a C57BL6 background, were created by CRISPR-based mutagenesis and homologous recombination using donor DNA composed of cloned human DNA containing the c.4886+1249(G>T) mutation (45) flanked by mouse sequences to promote DNA replacement in the targeted mouse *Dysf* locus (Figure 1B and Supplemental Figure 1A). A 3,978 bp segment of human genomic DNA was amplified from myogenic cells derived from a patient carrying this mutation (patient 1) (45) using primers with cloning linkers targeting sequences (human sequence underlined) in exon 44 (forward primer *DYSF* g44-45 F-2, 5′-AGTGTGGTGGAATTCTCCAGTGTGATCCTTACATCAAGATCTCC-3′) and exon 45 (reverse primer *DYSF* g44-45 R-2, 5′-GACTCGAGCGGCCGCTACACACAGTAGGTCTGTGGGAGTC-3′) and high-fidelity PCR reagents (Phusion Hi-Fidelity DNA polymerase, New England Biolabs). The amplified DNA segment was cloned into plasmid pcDNA3, and the sequence and presence of the intronic mutation were verified by Sanger sequencing. Mouse DNA flanking the 5′ (908 bp) and 3′ (925 bp) regions of exons 44–45 were synthesized (gBlocks, IDT) and ligated to the human DNA segment to create a mouse-human-mouse donor construct using NEBuilder HiFi DNA Assembly reagents (New England Biolabs). Mutant mice carrying this human construct in place of the endogenous *Dysf* sequence were produced by Biocytogen. For this step, to facilitate recombination into the mouse locus, the donor DNA construct was modified to include additional mouse *Dysf* flanking the DNA sequence on both sides of the human DNA; the final donor DNA had 1,491 bp flanking the 5′ end of exon 44 and 1,367 bp flanking the 3′ end of exon 45 (Supplemental Figure 1A). The donor construct was injected into C57BL/6J mouse oocytes along with guide RNAs and CRISPR/Cas9 reagents to induce site-specific host DNA cleavage and integration of the mouse-human-mouse donor DNA by homologous recombination. Founder mice were screened for successful donor integration using diagnostic PCR analysis (Supplemental Table 2) followed by Southern blot analysis and Sanger sequencing. RT-PCR was performed on RNA from mutant mouse muscles using primers targeting mouse exon 43 and 47 that flank the DNA donor integration site (mEx43-F1 5′-CTCCCAGAAGATCCAGCCATC-3′; mEx47-R1 5′-ACATGCAGGGCTAAGCGTT-3′), and then Sanger sequencing was performed on RT-PCR products. Additional primers used for RT-PCR analysis are in Supplemental Table 3. Conditions for routine genotyping are listed in Supplemental Table 1. Mice were backcrossed to C57BL/6 (The Jackson Laboratory, strain 000664) or bred as heterozygotes or homozygotes to generate desired genotypes for studies. Both male and female mice were used for studies.

### Behavior.

Grip strength was monitored using the Mark-10 Digital Force Gauge. The strength of both front limbs and all 4 limbs of mice were measured. The mice were manually held up to the wire mesh platform by their tail and allowed to grip it with their front paws or all 4 paws. The mice were then gently pulled back by hand to measure their peak strength. Three trials were taken per mouse and averaged to get a single measurement.

### Oligonucleotide synthesis.

Unconjugated and 5′ cholesterol–conjugated ASOs were synthesized on a Dr. Oligo 48 DNA/RNA synthesizer (Biolytic) at a 1 μmol scale using universal UNY Linker support (1000Å LCAA-CPG; ChemGenes) (Supplemental Table 4). For cholesterol conjugation, 5′-CholTEG phosphoramidite (ChemGenes) was incorporated during the final synthetic cycle according to the manufacturer’s instructions. All 2′-O-methoxyethyl–modified phosphoramidites were coupled for 8 minutes per cycle. Upon completion of synthesis, the solid support was treated with concentrated aqueous ammonia (30% w/w) and incubated at 55°C for 16 hours to achieve base deprotection and cleavage from the support. The crude ASOs were characterized by liquid chromatography–mass spectrometry (LC-MS) using an Agilent Q-TOF system equipped with an AdvanceBio Oligonucleotide C18 column (2.1 × 50 mm). For desalting, the oligonucleotide solutions were subjected to diafiltration using 3 kDa Amicon centrifugal filters with 1 wash in 1× PBS followed by 3 washes in nuclease-free water to remove salts and low-MW impurities. Crude cholesterol-conjugated oligonucleotide was further purified by reversed-phase HPLC (RP-HPLC) to remove truncated sequences and unconjugated material. The purified oligonucleotides were collected, desalted again by diafiltration, and quantified by UV absorbance at 260 nm. Cholesterol-conjugated oligonucleotides were hybridized to the PMO to create the HDOs using methods similar to those in a previous study (59).

### PPMO conjugation and purification.

A PMO bearing a bicyclo[6.1.0]nonyne (BCN) group at the 3′ end (synthesized by Gene Tools) was conjugated to a cell-penetrating peptide (R(6-aminohexanoic acid)RR(β-alanine)RRFQILYR(β-alanine)R(6-aminohexanoic acid)R(β-alanine)VGGGG) (KAzide)); KAzide is lysine containing an azido moiety (synthesized by Biosynth) via copper-free strain-promoted azide-alkyne cycloaddition. The resulting PPMO conjugate (PPMO^PE44.1^) was purified by RP-HPLC and analyzed by LC-MS to confirm identity and purity. Final desalting and buffer exchange were performed by diafiltration using a 3 kDa MW cutoff Amicon Ultra centrifugal filter (MilliporeSigma), with 1 wash in 1× PBS followed by 3 washes in deionized water.

### In vitro assay of exon skipping in patient-derived myotubes.

Dermal fibroblast cultures derived from patients were converted into myotubes by an inducible system as previously described (45). The resulting myogenic cells were plated in growth media at 1,000,000 per well in a 6-well tissue culture plate and allowed to differentiate into myotubes for 7 days. ASOs modified with 2′-O-methoxyethyl and phosphorothioate backbones were diluted in OptiMEM (Gibco) to a stock concentration 20 times the working concentration (200 mM, 100 mM, 60 mM, 20 mM, 15 mM, 5 mM, and 0.2 mM), mixed 1:1 with Lipofectamine RNAiMAX Transfection reagent (Invitrogen, 13778150), and then applied to myotubes. Cells were incubated for 72 hours at 37°C and 5% CO_2_. PMOs and a nontargeting PMO were added directly to the plated cells, diluting them 20-fold, followed by adding 6 mL of EndoPorter per 1 mL of media (Gene Tools).

### i.v. injections.

Three-month-old homozygous PE44.1 mice were used in these experiments. PMO and PMO HDO conjugates were prepared in a saline solution with the final doses of 80 mg/kg for the PMO and PMO HDO. PPMO conjugate was prepared in a saline solution at a final dose of 12.5 mg/kg. The dosing for these compounds followed representative published reports; we selected a low-end dose of the peptide because of its known potential renal toxicity (60, 79, 80). The drug was administered once via the tail vein in awake mice. One week after treatment, blood was collected for serum, and then tissues were frozen for 30 seconds in 2-methylbutane cooled in liquid nitrogen and then stored at –80°C.

### Measuring mRNA expression.

For reverse transcription, 2,000 ng of total RNA was used with the High-Capacity cDNA reverse transcription kit (Thermo Fisher Scientific) according to the manufacturer’s protocol. For PCR reactions, the following primers were used: DYSF 44.1 forward: 5′-TAACTACATCCCCTGCACGC-3′ and DYSF 44.1 reverse: 5′ -CGACCGTCTCACCGATCTTT-3′. The cycle conditions were as follows: 94°C for 5 minutes, followed by 30 cycles of 20 seconds at 94°C, 20 seconds at 55°C, and 1 minute at 68°C. For qPCR, individual reactions were performed using the following volumes: 1 mL of the reverse transcription reaction mixture (20 ng cDNA total), 5 mL of Luna Universal qPCR Master Mix (New England Biolabs M3003), 0.025 mL of a 100 mM solution of a forward primer, 0.025 mL of a 100 mM solution of a reverse primer, and 3.95 mL of water for a final reaction volume of 10 uL. The qPCR reactions were conducted in technical triplicates on a CFX384 Real Time System (BioRad). The cycle conditions were as follows: 94°C for 15 minutes, followed by 49 cycles of 10 seconds at 94°C, 30 seconds at 58°C, and 30 seconds at 72°C. All qPCR was done using a fluorescent binding dye of double-stranded DNA, and gene expression was calculated using the ΔΔCT method normalized to the TATA-box binding protein (TBP) housekeeping gene with the following primers: *Dysf*-PE44.1 forward: 5′-AGTGAGGACCCAGTCTCCTT-3′, *Dysf*-PE44.1 reverse: 5′-AGCTCGAACATCCTCCAATCC-3′, DYSF mouse WT forward: 5′-GCCTGTATTCGGAAAGATGT-3′, DYSF mouse WT reverse: 5′-AATCTTCTCATCCTTGGAGA-3′, DYSF human WT forward: 5′-CCGTCTCACCGATCTTTTCG-3′, DYSF human WT reverse: 5′-GCCCGTATTTGGAAAGATGT-3′, TBP human housekeeping forward: 5′-GAAGTCCAAGAACTTAGCTG-3′, TBP human housekeeping reverse: 5′-GCCAAGAGTGAAGAACAG-3′, TBP mouse housekeeping forward: 5′-ACTTGACCTAAAGACCATTGCACTT-3′, and TBP mouse housekeeping reverse: 5′-TCTTCCTAACACGCTGGTCAAA-3′.

### IHC.

For histopathology analysis, psoas, gluteus, gastrocnemius, and anterior tibialis were dissected from 4-, 8-, and 12-month-old pseudoexon 44.1 dysferlin mice and WT littermates. Muscles were frozen for 30 seconds in 2-methylbutane cooled in liquid nitrogen. Samples were then stored at –80°C. Transverse muscle sections were taken at 10 mm and collected directly onto super frost plus glass slides using a CM1950 cryostat (Leica Biosystems). H&E staining on frozen muscle sections was performed as previously described (81). For dysferlin visualization, tissue sections were labeled with rabbit anti-dysferlin (Romeo, Abclonal, A19572) at 1:100 in blocking solution (10% donkey serum, 0.1% Triton X-100 in TBS [TBST]) overnight at 4°C in a humid chamber. Sections were washed 3 times for 5 minutes with TBST, and then incubated with an Alexa Fluor 488 donkey anti-rabbit antibody (Invitrogen, A21206) at 1:250 dilution and DAPI at 1:3,000 dilution (Sigma-Aldrich, D9542) to stain nuclei. Images were acquired using the Nikon Eclipse Ti at ×20 original magnification and analyzed using Fiji ImageJ (NIH) and NIS Elements AR Analysis software. The same protocol was used for F4/80 (primary rat-anti F4/80, ProSci, 76-052), 1:100 in TBST; secondary donkey anti-rat (Invitrogen, A21209), 1:250 in TBST; CD45 (primary rabbit-anti CD45, Abcam, ab40763), 1:100 in TBST; secondary donkey anti-rabbit (Invitrogen, A21206); CD8 (BD Pharmingen, 558733), 1:250 in TBST; secondary donkey anti-rat (Invitrogen, A21209); CD11b (BD Pharmingen, 553308), 1:250 in TBST; secondary donkey anti-rat (Invitrogen, A21209) 1:250 in TBST, and PDGFα (ProSci, 59-024), 1:50 in TBST; and secondary donkey anti-rabbit (Invitrogen, A21206) 1:250 in TBST.

### Protein extraction and immunoblotting.

Western blotting was carried out as previously described (45). Briefly, muscle tissue was homogenized in RIPA buffer (40 mmol/L Tris-HCL pH 8, 150 mmol/L sodium chloride 1% Triton X-100, 0.5% sodium deoxycholate, 0.5% SDS) with protease inhibitors (Roche). The protein sample was quantified using a bicinchoninic acid (BCA) protein assay (Thermo Fisher Scientific, 23250). Samples (5 μg/lane) were heated at 95°C for 5 minutes in tris-glycine SDS supplemented with 5% 2-mercaptoethanol, and then separated on a 4%–12% gradient tris-glycine gel (Invitrogen). Proteins were transferred to nitrocellulose paper using the iBlot 2 device (Invitrogen). Blots were then incubated overnight in Intercept blocking buffer (Licor) containing mouse anti-DYSF antibody (NCL Hamlet, 1:1,000, and goat anti–β-actin antibody (Abcam, ab8229, 1:2,000). Blots were washed in PBS supplemented with 0.1% Tween-20 and incubated for 1 hour with infrared antibodies (donkey anti-mouse 800CW 926-32212, 1:6,000 and donkey anti-goat 680RD 926-68074 1:6,000) for 1 hour at room temperature, and then imaged using an Odyssey infrared imager (Licor) and quantified using Fiji imaging software with β-actin as a protein normalization control.

### Functional assays of membrane permeation.

First, 4-, 8-, and 12-month-old homozygous PE44.1 mice, age-matched with heterozygous and WT littermates, were i.p. injected with a 10 mg/mL solution of Evans blue dye (Sigma-Aldrich) dissolved into PBS at 5 mL per gram of mouse body weight. Muscles were harvested 24 hours after injection, weighed, and incubated in 1 mL of formamide (MilliporeSigma) at 55°C for 2 hours. Then, 200 mL of the solvent was collected and the absorption was measured at 640 nm (51). For serum CK, 10 mL of serum collected from animals was used with the Liquid Creatine Kinase reagent set (Pointe) according to the manufacturer’s protocol.

### Toxicity evaluation.

Mice were treated with PPMO^PE44.1^ at low (4.2 mg/kg), medium (12.5 mg/kg), and high (37.5 mg/kg) doses for 24 hours, and serum samples were sent to IDEXX Laboratories for evaluation of renal and liver toxicity markers.

### Plate-based oligo electrochemiluminescent ELISA for PMO quantification.

PMOs were spiked into tissue homogenate and homogenized in PBS or serum at a designated standard curve range prepared at 4 to 2,500 ng/mL. Standard curve or unknown samples were added to a 96-well PCR plate to a final volume of 50 mL. PMO sequence-specific capture and detection oligonucleotides (see below) were prepared in a hybridization buffer of 60 mM Na_2_PO_4_ (pH 7.0, dibasic), 1 M NaCl, 5 mM EDTA, and 0.02% Tween 20. A 2× mixture of probes in hybridization buffer was prepared at 20 nM, and 50 mL of this mixture was added to the PCR plate containing diluted standards and unknowns. The final mixture within the PCR plate resulted in a probe concentration of 10 nM and sample dilution value of 1:20. Sample and probes were hybridized on a thermal cycler under the following conditions: 90°C for 5 minutes, 40°C for 30 minutes, and a final hold at 12°C. After hybridization, 45 mL of samples were transferred to an MSD Gold 96-well Streptavidin SECTOR plate (Meso Scale Diagnostics) in duplicate and were incubated on a shaking platform for 30 minutes. After incubation, the plate was washed with prepared wash solution made up of 0.05% Tween-20 in TBS. After washing, plates were incubated for 1 hour with 50 mL of 0.5 mg/mL ruthenium-labeled anti-digoxygenin antibody in wash buffer with 2% BSA. After a final wash, 150 mL of 1× MSD Read buffer T (Meso Scale Diagnostics) was added, and the plate was read on an MSD Sector S 600 instrument (Meso Scale Diagnostics). A nonlinear regression analysis was performed to calculate the concentrations of reference compound from the signal intensities via interpolation from a calibration curve using a 4-parameter logistic (4PL) model (weighting factor = 1/Y^2^) in GraphPad Prism 10. The modified ASO probes are as follows (IDT) — ASO capture probe: 5′-/5Bio/GAGCTCCCACCA-3′ and ASO detection probe: 5′-AGGGATTGGA/3Dig_N/-3′; the underlined base is the LNA modification, 5Bio is biotin conjugation with C6 linker, and 3Dig_N is digoxygenin (NHS) ester.

### Statistics.

Data are expressed as the mean ± SEM (error bars) with statistical significance determined using 1- or 2-way ANOVA with post hoc Tukey’s tests. For this analysis, we used GraphPad Prism 10 statistical analysis software. *P* values of less than 0.05 were considered significant.

### Study approval.

All animal procedures were reviewed and approved by the IACUC at University of Massachusetts Chan Medical School and performed in compliance with all relevant ethical regulations.

### Data availability.

All data supporting the findings of this study are available within the article or from the corresponding author upon reasonable request. Values for all data points in graphs are reported in the Supporting Data Values file.

## Author contributions

RHB and JAD conceptualized the study. RHB, JAD, and JEG designed the research studies. JEG conducted experiments, acquired data, and analyzed data. RHB and JEG wrote the manuscript. GP, AW, and JKW provided chemistry expertise, chemical synthesis, and reagents. All authors reviewed and edited the manuscript. RHB supervised the study and acquired funding.

## Conflict of interest

The authors have declared that no conflict of interest exists.

## Funding support

- Cecil B. Day Foundation (to RHB).

## Supplementary Material

Supplemental data

Unedited blot and gel images

Supporting data values

## Version 1 07/14/2026

In-Press Preview

## Version 2 08/24/2026

Electronic publication
