# Supplementary material for: Peptide-phosphorodiamidate morpholino oligomer therapy for dysferlinopathy induces pseudoexon skipping and restoration of functional protein
Source: JCI Insight. 2026 Jul 14;11(16):e204742. doi: 10.1172/jci.insight.204742 (PMC13502175; doi:10.1172/jci.insight.204742)
Supplement: Supplementary file 1 [file jciinsight-11-204742-s074.pdf]

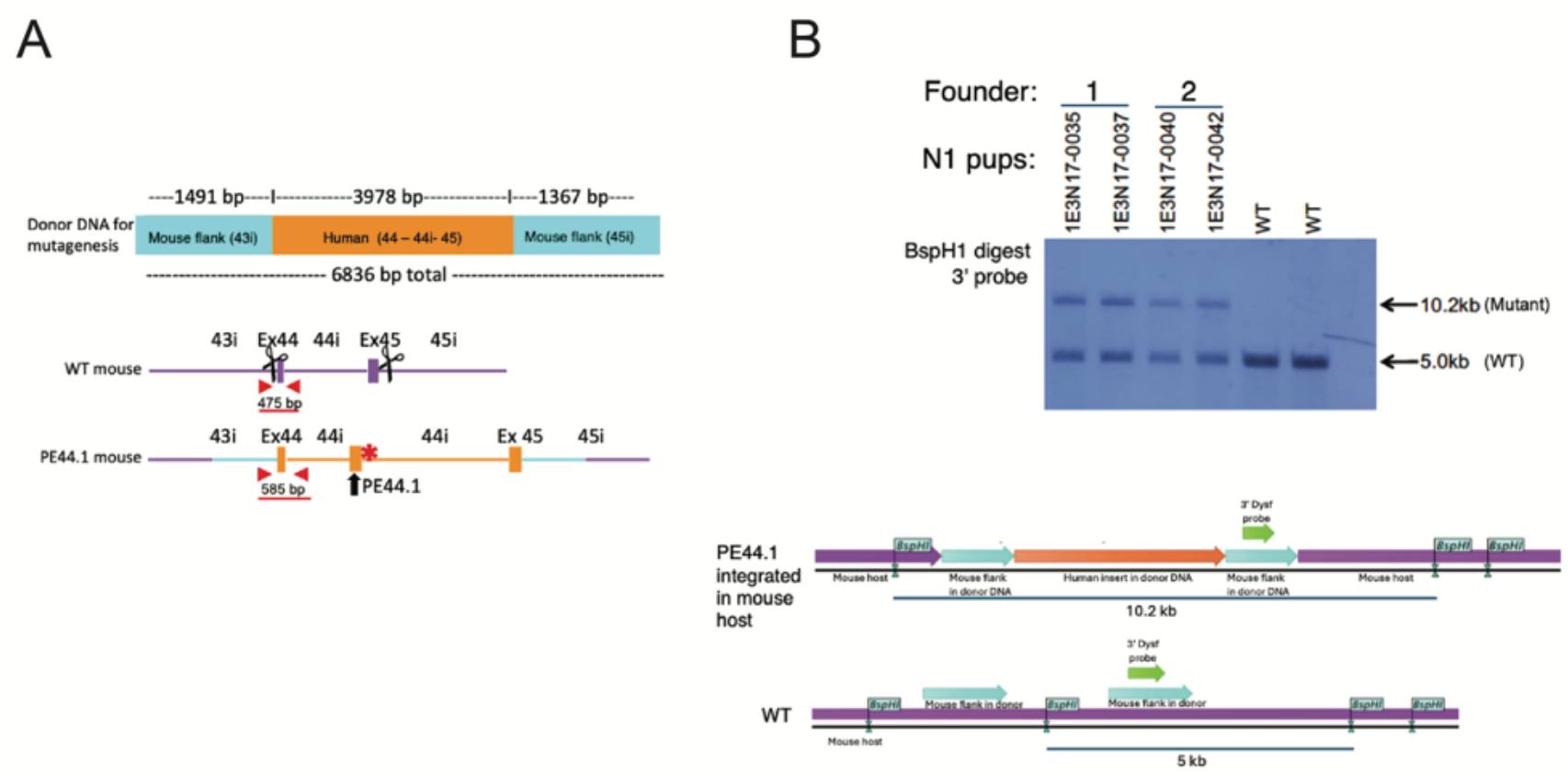

Supplemental Figure S1. DYSF-PE44.1 mice were created by CRISPR-Cas9-directed homologous recombination into the mouse Dysf locus. (A) The donor DNA construct used to create the mutant mice consisted of human DYSF DNA (exon 44-intron 44i- exon 45) flanked by mouse DNA arms to promote homologous recombination. Cas9 cleavage sites in the host mouse gene are indicated by scissors. The final PE44.1 mouse integrated sequence shows the human exons and PE44.1 integrated in the mouse locus, with the c.4886+1249(G>T) point mutation site indicated (star). Genotype-specific primers (arrowheads). (B) Southern blot of DNA from heterozygous N1 progeny of two founder lines shows the predicted mutant restriction fragment along with the wild-type WT fragment. There was no mutant fragment in WT mice. The blots were probed with a 506 bp 3’ Dysf probe indicated on the map.

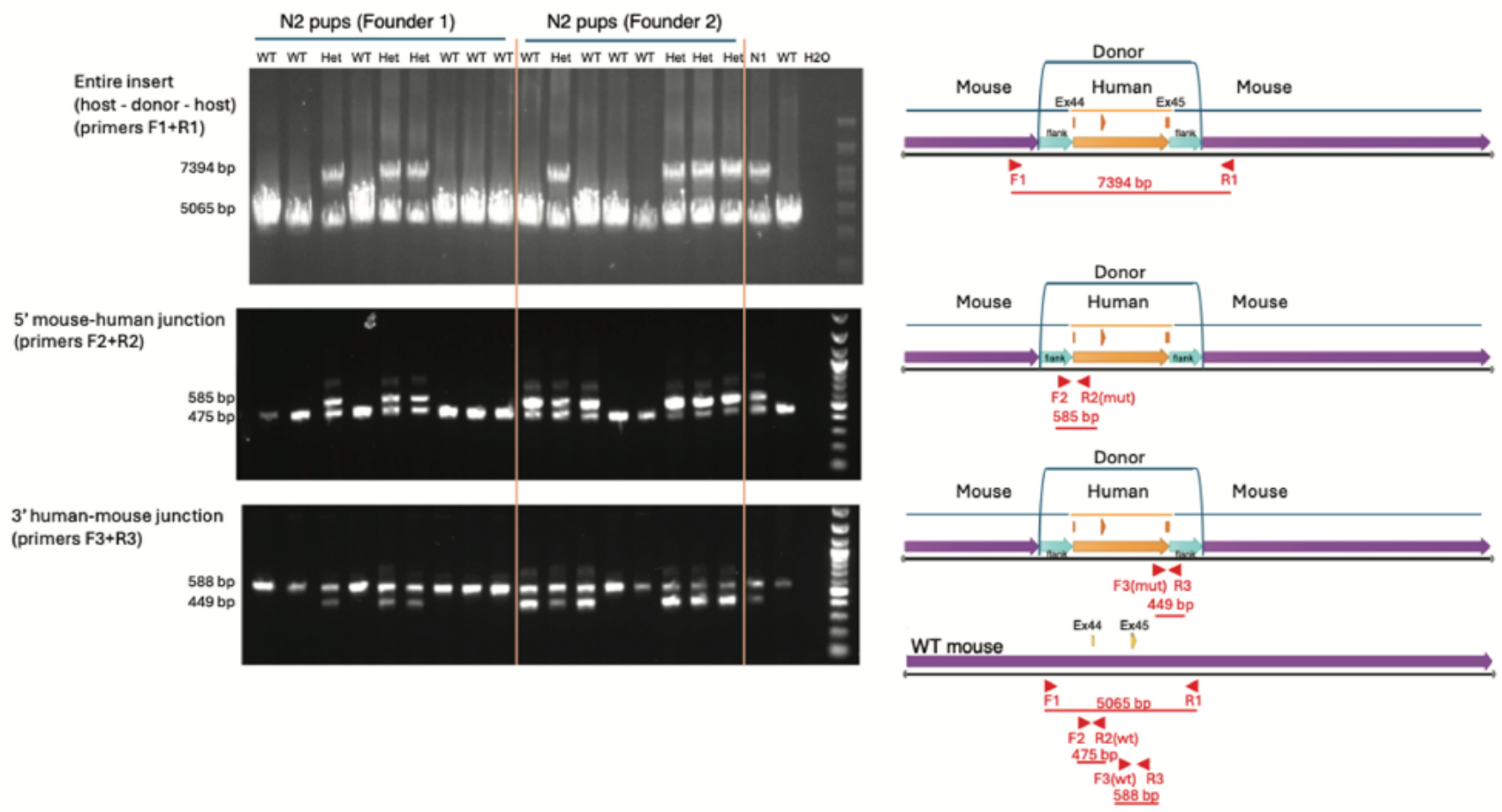

Supplemental Figure S2. Analysis of the integration of the mouse-human-mouse donor DNA into the mouse Dysf locus. Diagnostic primers were used in long or short-range PCR to amplify genomic DNA sequences that span the regions indicated on the maps shown. Mice heterozygous for the DYSF-PE44.1 allele exhibited the predicted size PCR products that span the entire integration site, the 5' mouse-human junction site, and the 3' human-mouse junction site. As heterozygotes, they each exhibited the predicted WT products.

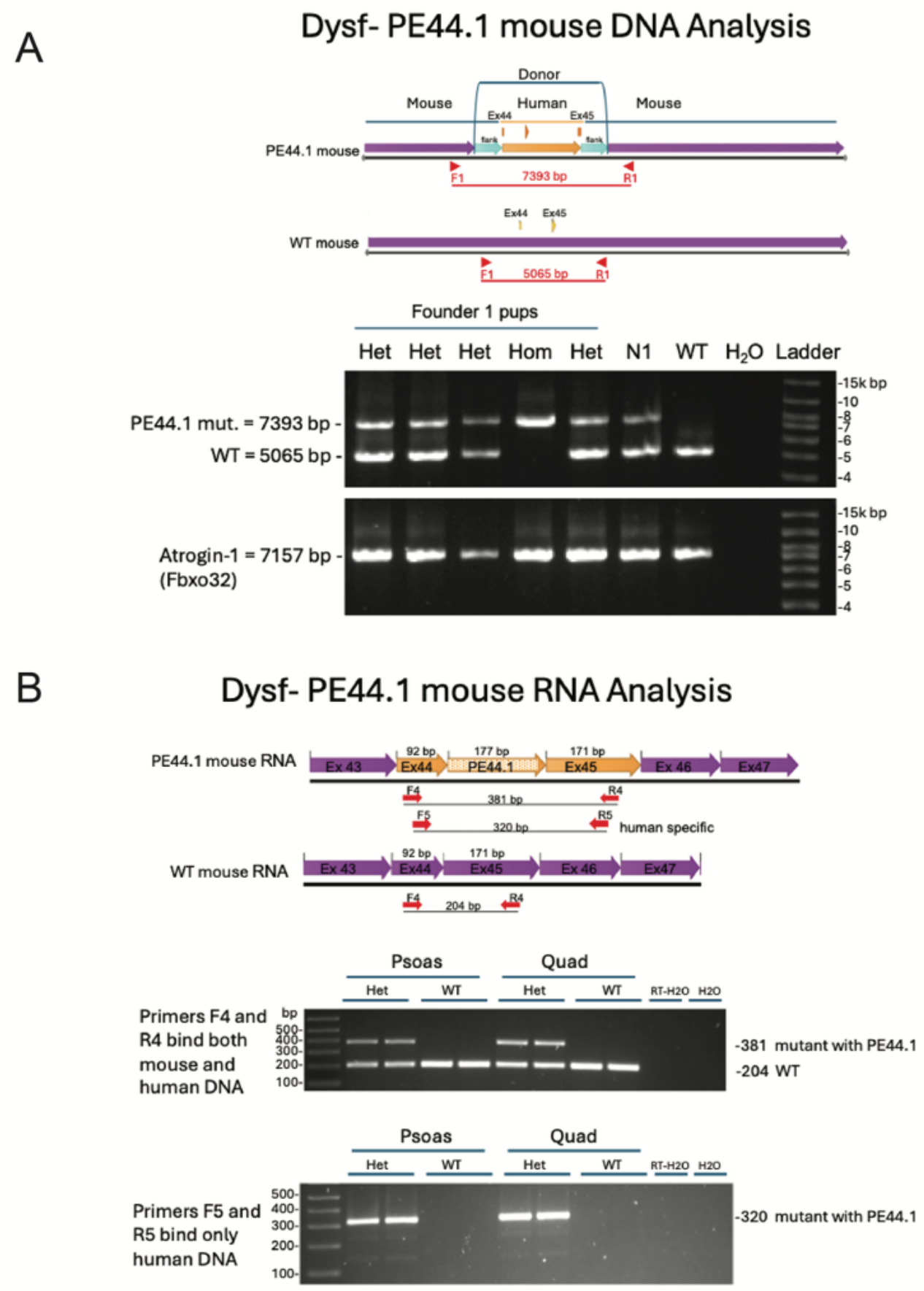

Supplemental Figure S3. (A) Additional long-range PCR demonstrates that only the mutant PCR amplicon is observed in homozygous mice. An unrelated long-range PCR product for atrogin-1 provides a control for DNA quality, sufficient for long-range amplification of all samples. (B) RNA analysis by RT-PCR demonstrates that heterozygous mutant mice express mRNA containing PE44.1, along with WT mRNA. Primers (arrowheads) are indicated on the maps showing the exon structure. N1, N1 het from founder line 1.

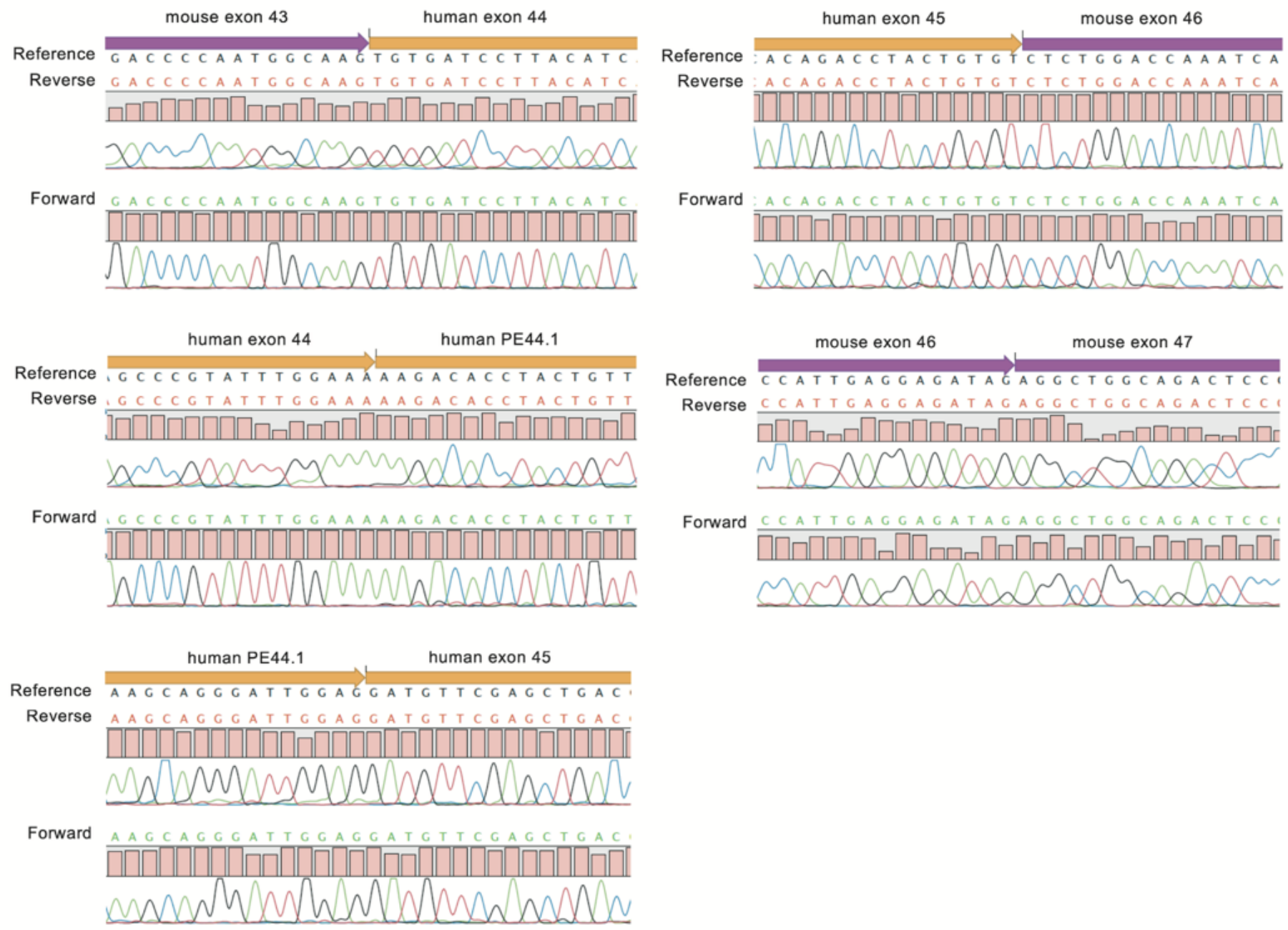

Supplemental Figure S4. Sequence analysis of homozygous PE44.1 muscle mRNA confirms the accurate splicing of mouse-human hybrid dysferlin mRNA and inclusion fo the human mutant PE44.1 sequence. RT-PCR was performed using mutant psoas muscle mRNA and PCR primers amplifying a region of mouse RNA from exon 43 to 47, flanking the mutation site. Shown here are sequences around each of the splice junctions, in both the reverse and forward orientation, aligned with the predicted reference sequence for the mouse-human-mouse mRNA.

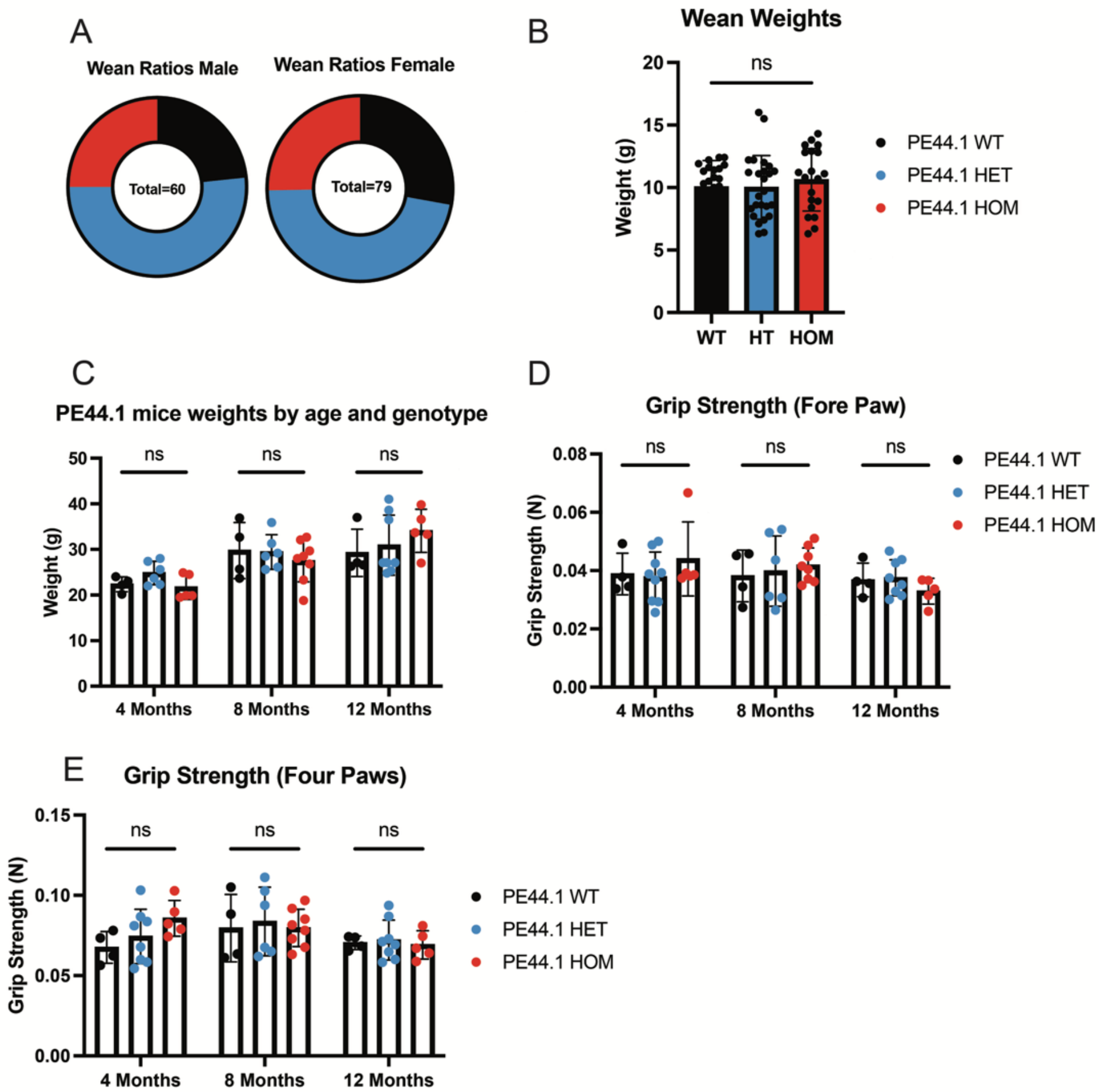

Supplemental Figure S5. Behavioral and breeding analysis of PE44.1 mutant mice (A) Wean ratios of heterozygous - heterozygous matings of PE44.1 mutant mice (n=139). (B) Wean weights of homozygous, heterozygous PE44.1 mutant mice, and wildtype littermates. (C) Body weights, (D) Four paw grip strength, (E) Fore paw grip strength of homozygous, heterozygous PE44.1 mutant mice, and wildtype littermates at 4, 8, and 12 months of age.

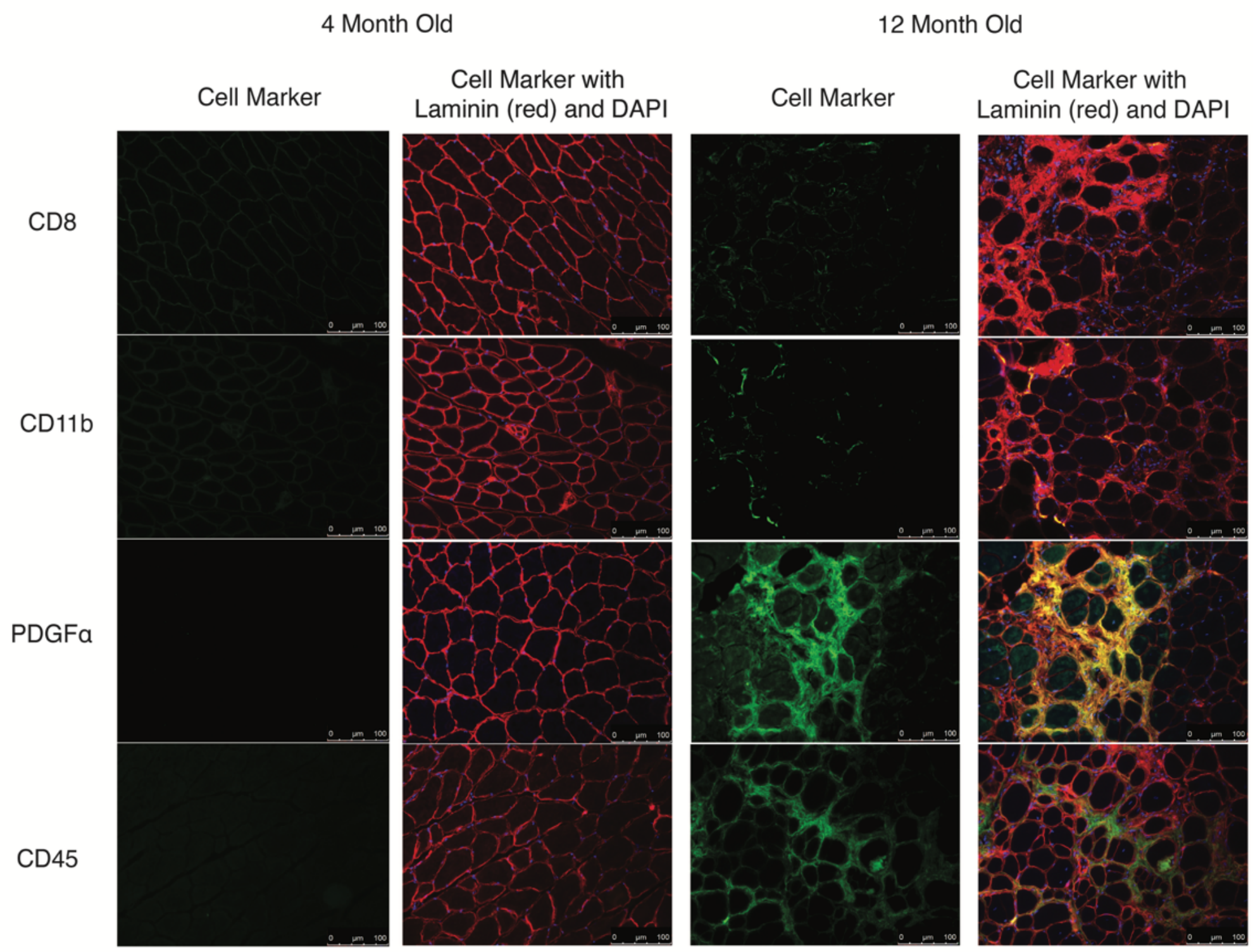

Supplemental Figure S6. Immunohistochemistry staining of non muscle cells in muscle tissue. First two columns are tissue of 4 month old mice gluteus muscle and last two columns are tissue of 12 month old animals gluteus muscle. Columns one and three show the cell marker (green) and column two and four show the cell marker combined with laminin (red) and dapi (blue). Row one is staining for CD8 (green), row two is staining for Cd11b (green), row three is stainig for PDGFα (green) and row four shows staining for CD45 (magenta). Scale bar showing 100 um.

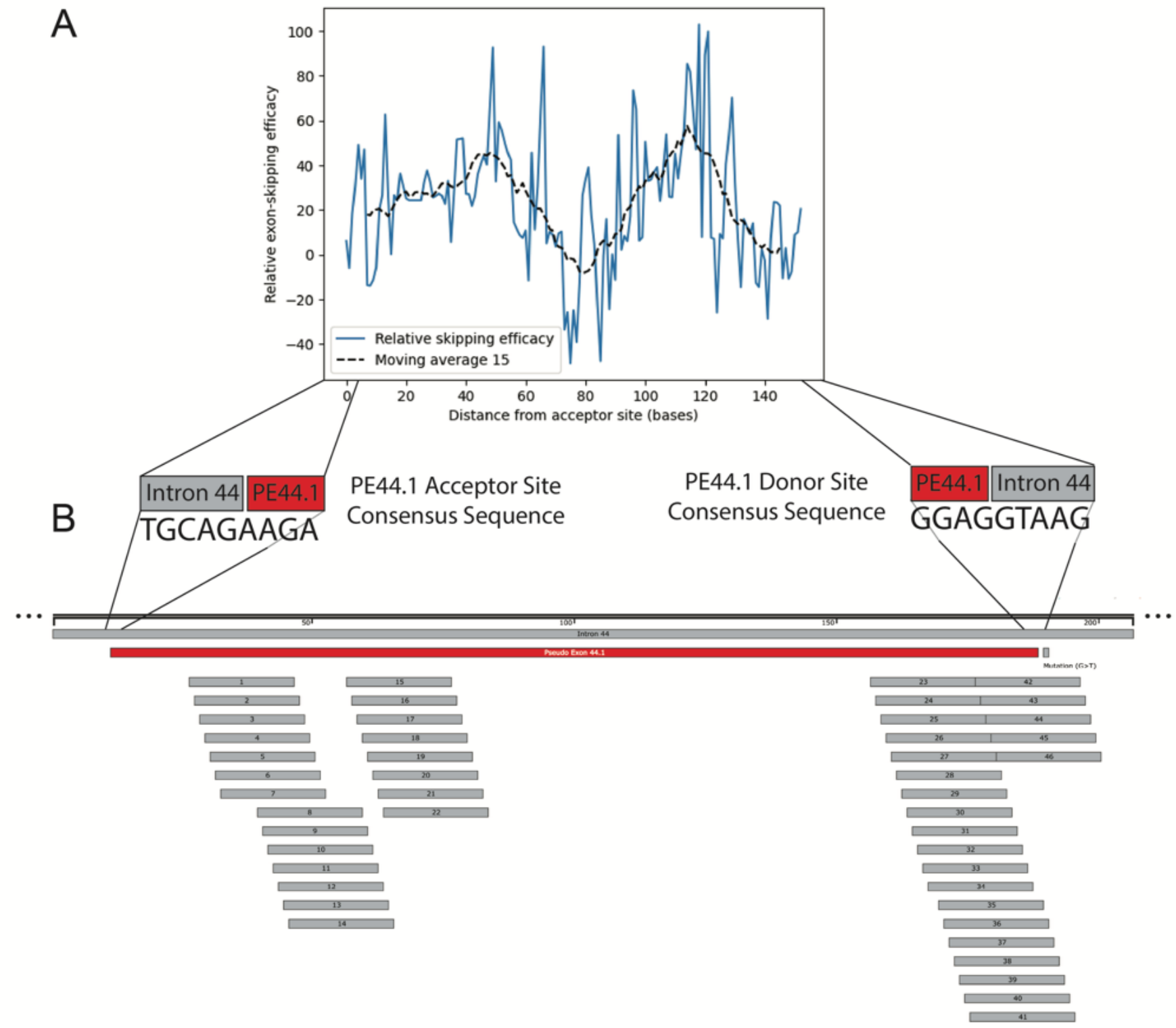

Supplemental Figure S7. Antisense oligonucleotides are designed to target critical sites within the pseudo exon. (A) In silico analysis of splicing regulatory elements on the pathogenic pseudo exon. Potential splicing enhancer regions were predicted by ESE Finder 3.0 eSkip Finder showing relative exon-skipping efficacy for ASO target sites. (B) Design strategy for ASOs against exon splice enhancer sites within the pseudo exon utilizing In silico analysis. The pseudo exon is shown in red.

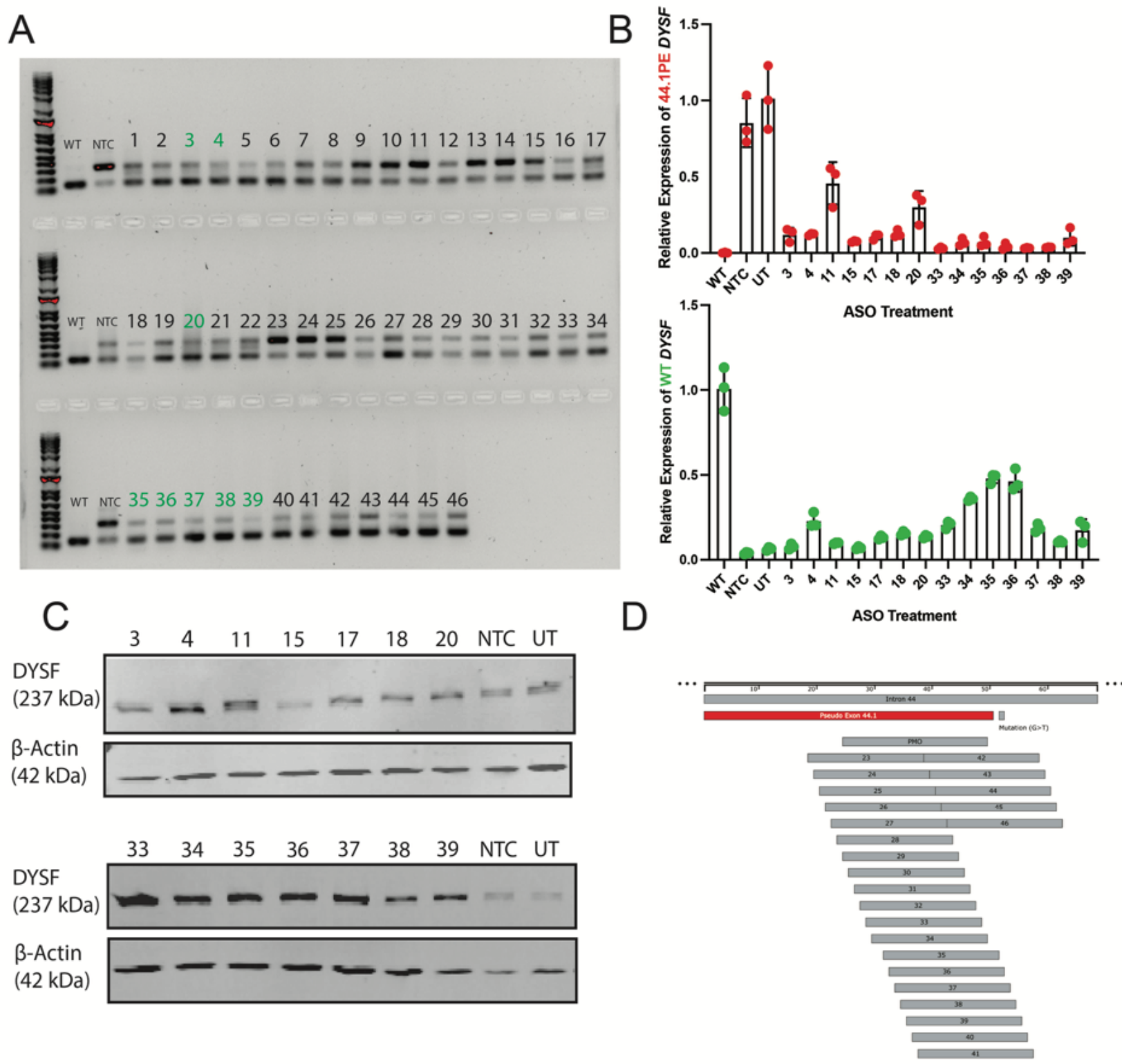

Supplemental Figure S8. Screening strategy for empirically determining exon splice enhancer sites for skipping the pseudoexon. (A) Initial screen of 46 ASOs targeting regions of high exon splice enhancer site binding evaluated through RT-PCR of mRNA from patient derived myogenic cells treated with 1 uM ASOs for 72 hrs. Two bands shown, upper band indicates the inclusion of the pseudoexon while the lower band indicates no pseudoexon. (B) Select ASOs (numbered in green in A) were further evaluated with RT-qPCR measuring the expression of both the pseudoexon-containing transcripts and the wildtype transcripts. (C) Western blot detecting dysferlin and β-actin of patient derived myotubes that are heterozygous for PE44.1 mutation treated with select ASOs. (D) Diagram illustrating site of ASOs with high success of skipping pseudoexon 44.1 in patient derived myotubes and the PMO designed against this site.

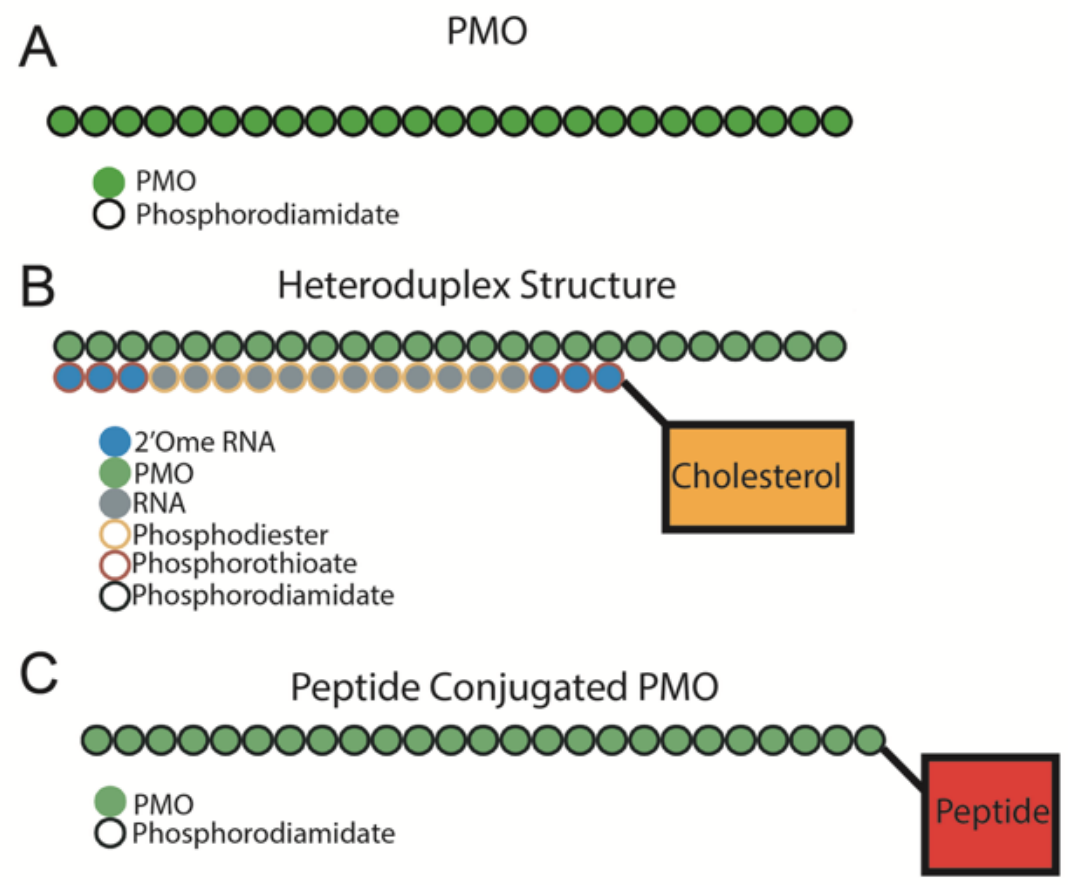

Supplemental Figure S9. Oligo chemistry and structure. (A,B,C) Structural and chemical make up of (A) PMO, (B) Heteroduplex, and (C) PPMO.

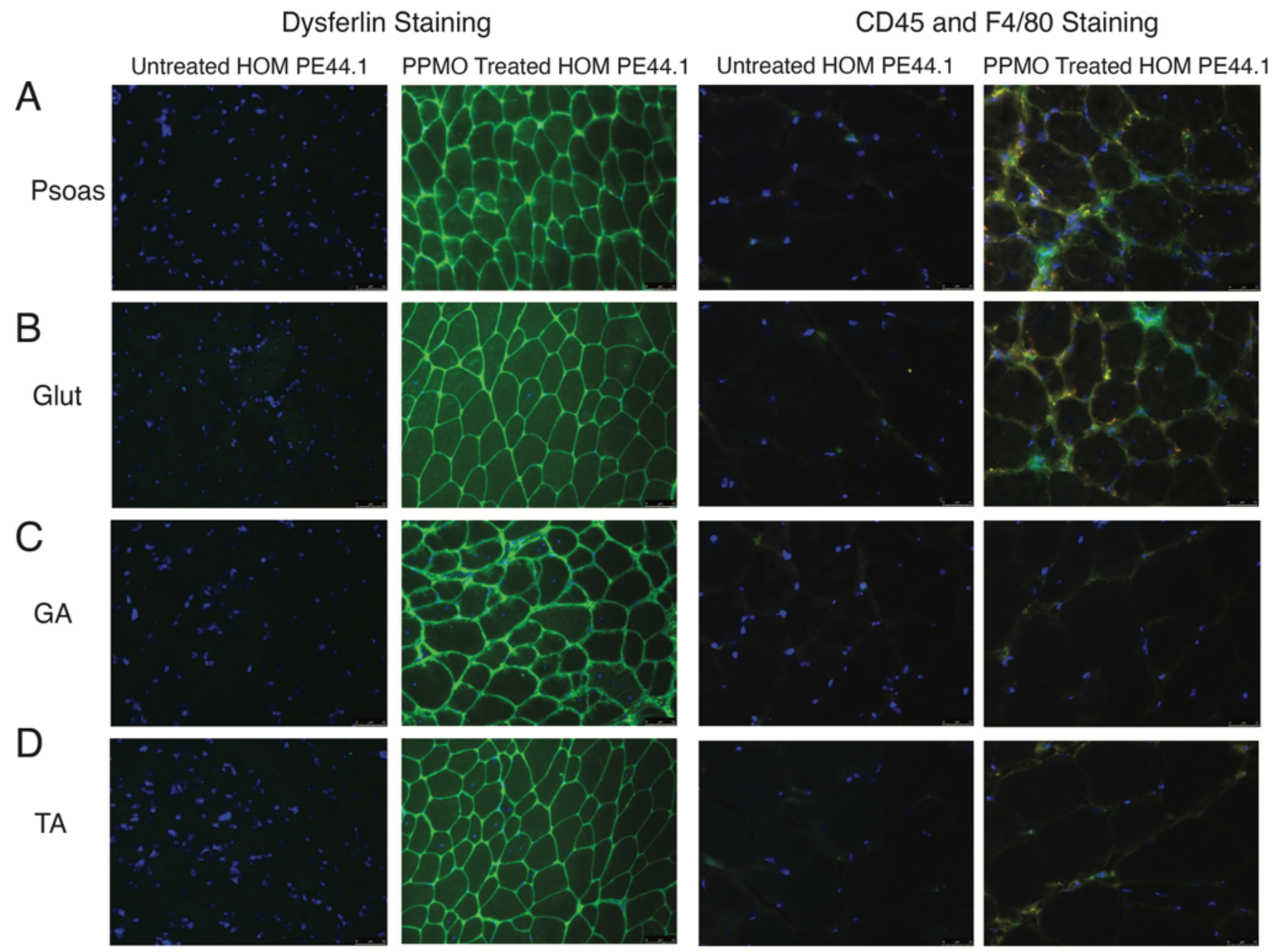

Supplemental Figure S10. Representative tissue images of (A) Psoas, (B) Glut, (C) Gastrocnemius, and (D) Anterior Tibialis of untreated and PPMO treated mice with DAPI in blue and Dysferlin in green. Staining for immuno reactivity CD45 in green and F4/80 in red.

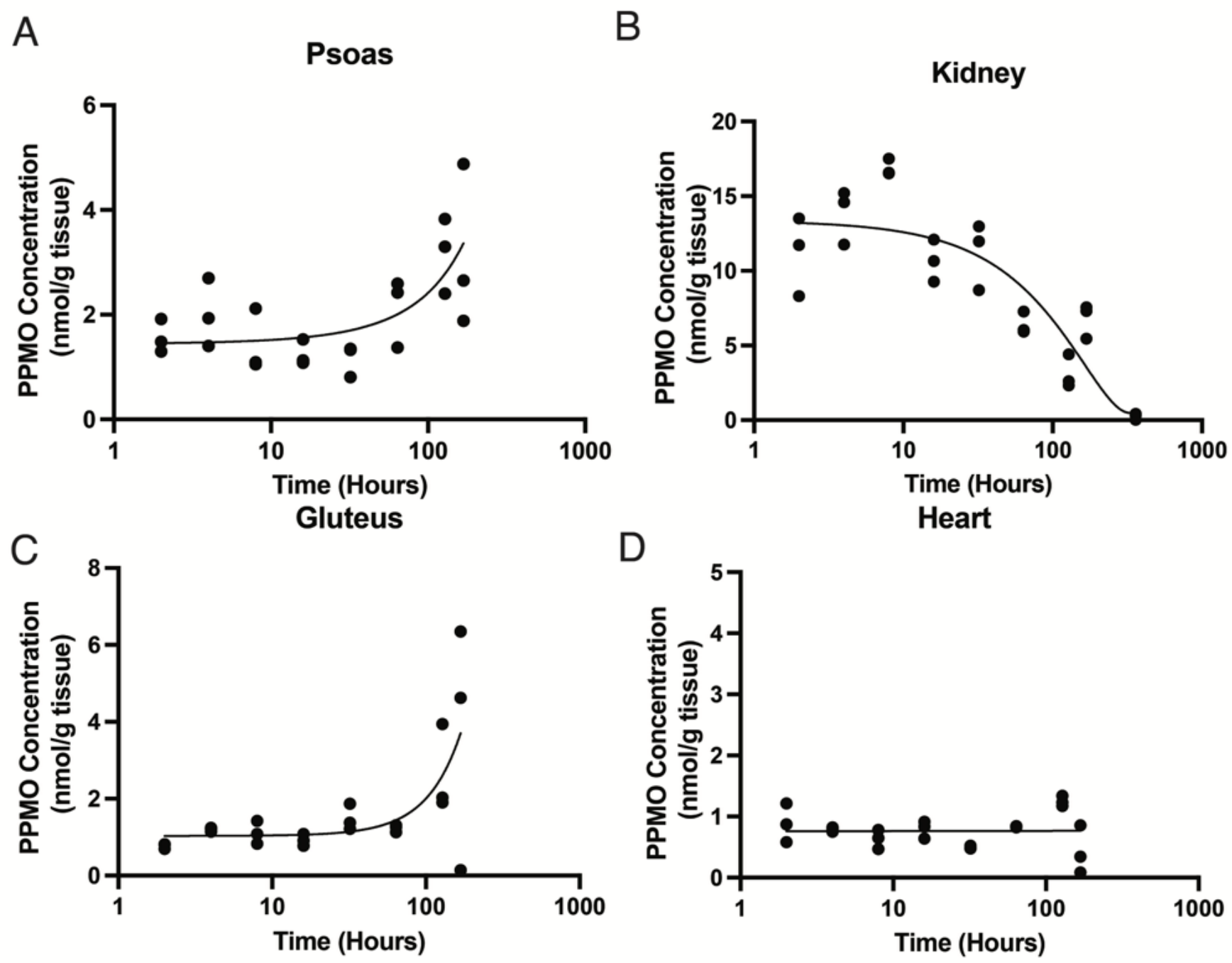

Supplemental Figure S11. Uptake and clearance of PPMOPE44.1 into tissue overtime (nM/g of tissue) in (E) kidney, (F) gluteus, (G) psoas, and (H) heart measured through a plate based oligonucleotide electro chemiluminescent immunoassay quantifying nucleic acids.

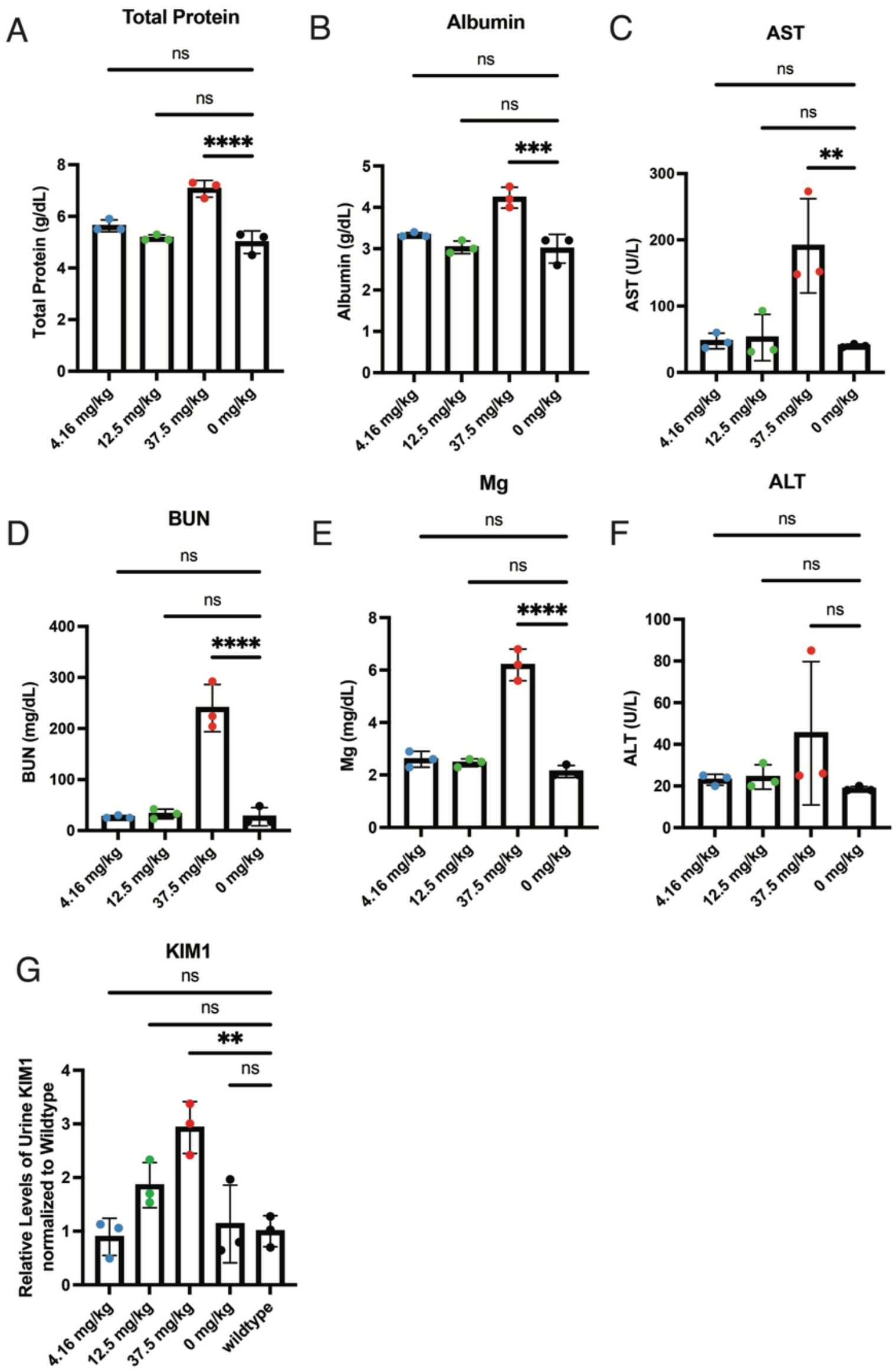

Supplemental Figure S12. Toxicology of mice treated with PPMOPE44.1 after 48 hours, evaluated by serum levels of common markers of kidney and liver function. (A) Total protein,(B) Albumin, (C) Aspartate Aminotransferase (AST), (D) Blood Urea Nitrogen (BUN), (E) Magnesium Ions, (F) Alanine Transaminase (ALT), (G) KIM1 levels in urine from treated mice normalized to untreated wildtype animals with hydration level accounted for through total protein concentration in the urine. Statistical differences were determined using one-way ANOVA and indicated as follows: * P 0.05, ** P 0.005, *** P 0.0002, and **** $P < 0.0001$.

Supplemental Table S1. PCR Primers for genotyping mutant Dysf-PE44.1 mice.

Primer Name	Primer Sequence (5' – 3')	Amplicon: WT	Amplicon; Mut
mDysf 43i-F<br>mDysf 44i-R<br>hDYSF 44i-R	ATGAGGGTAGGGGTCCAACA<br>CCTCTACCCGAAACCCAAGG<br>GCCCCCATAGACATGCAAGATT	475 bp	585 bp
Reagents and conditions: NEB One Taq Hot Start DNA Polymerase (New England Biolabs)<br>94°C, 30 sec<br>(94°C, 20 sec; 55°C, 20 sec; 68°C,1 min) x 30 cycles<br>68°C, 5 min<br>4°C hold			

Supplemental Table S2. PCR Primers for mouse genomic integration DNA analyses

	Primer label	Primer Name	Primer Sequence (5' – 3')	Amplicon WT	Amplicon Mut
Amplify entire insert	F1 (common)<br>R1 (common)	mDysf Lflk-F1<br>mDysf Rflk-R1	TCCCCTCCCAGAAGATCCAG<br>TGCCTGAAATCGTGCCTTCT	5065 bp	7393 bp
5' end (mouse -human junction)	F2 (common)<br>R2 (wt)<br>R2 (mut)	mDysf 43i-F<br>mDysf 44i-R<br>hDYSF 44i-R	ATGAGGGTAGGGGTCCAACA<br>CCTCTACCCGAAACCCAAGG<br>GCCCCCATAGACATGCAAGATT	585 bp	475 bp
3' end (human -mouse junction)	F3 (mut)<br>F3 (wt)<br>R3 (common)	PE44.1 geno-F3<br>Dysf-SJL F<br>Dysf-SJL R	TTGGCTGACATCGGGAATC<br>TACAGCTGCTCTGTGGGTGG<br>GTGCTGAGAATCAGGGTGGC	449 bp	588 bp
Non-related gene (Fbxo32) with long amplicon	Atrogin-1 F<br>Atrogin-1 R	Atrogin-1 F<br>Atrogin-1 R	TGGGTGTATCGGATGGAGAC<br>TCAGCCTCTGCATGATGTTC	7157 bp	7157 bp
Reagents and conditions: (for amplicons >3 kb)<br>NEB LongAmp HS DNA Polymerase (New England Biolabs)<br>94°C, 30 sec<br>(94°C, 20 sec; 59°C, 20 sec; 65°C, 7 min) x 30 cycles<br>65°C, 5 min<br>4°C hold					
Reagents and conditions for amplicons < 3 kb as in Supplemental Table 1S for genotyping.					

Supplemental Table S3. PCR Primers for Dysf-PE44.1 RNA analyses.

Primer	Primer Name	Primer Sequence (5' – 3')	Amplicon size: WT	Amplicon size: Mut
F4	DYSF44/45-F1	AGGGAAGAAATCAGTGAGTGACC	204 bp	381 bp
R4	DYSF44/45-QR	CCAAACTTGGACAGCAGCCT		
F5	DYSF44/45-F	TAACTACATCCCCTGCACGC	None	320 bp
R5	DYSF44/45-R	CGACCGTCTCACCGATCTTT		
Reagents and conditions: NEB One Taq Hot Start DNA Polymerase (New England Biolabs)<br>94°C, 30 sec<br>(94°C, 20 sec; 58°C, 20 sec; 68°C,1 min) x 30 cycles<br>68°C, 5 min<br>4°C hold				

Supplemental Table S4. Oligonucleotide List

ASO ID	Lab ID	Oligonucleotide Sequence
1	W-2158	(eU)#(eU)#(eA)#(eU)#(eA)#(eA)#(eG)#(eU)#(eG)#(eC)#(eA)#(eA)#(eU)#(eA)#(eU)#(eG)#(eU)#(eG)#(eA)#(eC)
2	W-2159	(eU)#(eU)#(eU)#(eA)#(eU)#(eA)#(eA)#(eG)#(eU)#(eG)#(eC)#(eA)#(eA)#(eU)#(eA)#(eU)#(eG)#(eU)#(eG)#(eA)
3	W-2160	(eG)#(eU)#(eU)#(eU)#(eA)#(eU)#(eA)#(eA)#(eG)#(eU)#(eG)#(eC)#(eA)#(eA)#(eU)#(eA)#(eU)#(eG)#(eU)#(eG)
4	W-2161	(eG)#(eG)#(eU)#(eU)#(eU)#(eA)#(eU)#(eA)#(eA)#(eG)#(eU)#(eG)#(eC)#(eA)#(eA)#(eU)#(eA)#(eU)#(eG)#(eU)
5	W-2162	(eC)#(eG)#(eG)#(eU)#(eU)#(eU)#(eA)#(eU)#(eA)#(eA)#(eG)#(eU)#(eG)#(eC)#(eA)#(eA)#(eU)#(eA)#(eU)#(eG)
6	W-2163	(eC)#(eC)#(eG)#(eG)#(eU)#(eU)#(eU)#(eA)#(eU)#(eA)#(eA)#(eG)#(eU)#(eG)#(eC)#(eA)#(eA)#(eU)#(eA)#(eU)
7	W-2164	(eC)#(eC)#(eC)#(eG)#(eG)#(eU)#(eU)#(eU)#(eA)#(eU)#(eA)#(eA)#(eG)#(eU)#(eG)#(eC)#(eA)#(eA)#(eU)#(eA)
8	W-2165	(eA)#(eA)#(eC)#(eU)#(eC)#(eA)#(eA)#(eC)#(eA)#(eG)#(eA)#(eC)#(eC)#(eC)#(eC)#(eG)#(eG)#(eU)#(eU)#(eU)
9	W-2166	(eU)#(eA)#(eA)#(eC)#(eU)#(eC)#(eA)#(eA)#(eC)#(eA)#(eG)#(eA)#(eC)#(eC)#(eC)#(eC)#(eG)#(eG)#(eU)#(eU)
10	W-2167	(eC)#(eU)#(eA)#(eA)#(eC)#(eU)#(eC)#(eA)#(eA)#(eC)#(eA)#(eG)#(eA)#(eC)#(eC)#(eC)#(eC)#(eG)#(eG)#(eU)
11	W-2168	(eA)#(eC)#(eU)#(eA)#(eA)#(eC)#(eU)#(eC)#(eA)#(eA)#(eC)#(eA)#(eG)#(eA)#(eC)#(eC)#(eC)#(eC)#(eG)#(eG)
12	W-2169	(eA)#(eA)#(eC)#(eU)#(eA)#(eA)#(eC)#(eU)#(eC)#(eA)#(eA)#(eC)#(eA)#(eG)#(eA)#(eC)#(eC)#(eC)#(eC)#(eG)
13	W-2170	(eC)#(eA)#(eA)#(eC)#(eU)#(eA)#(eA)#(eC)#(eU)#(eC)#(eA)#(eA)#(eC)#(eA)#(eG)#(eA)#(eC)#(eC)#(eC)#(eC)
14	W-2171	(eA)#(eC)#(eA)#(eA)#(eC)#(eU)#(eA)#(eA)#(eC)#(eU)#(eC)#(eA)#(eA)#(eC)#(eA)#(eG)#(eA)#(eC)#(eC)#(eC)
15	W-2172	(eA)#(eC)#(eU)#(eC)#(eA)#(eA)#(eC)#(eA)#(eG)#(eA)#(eC)#(eC)#(eC)#(eC)#(eG)#(eG)#(eU)#(eU)#(eU)#(eA)
16	W-2173	(eA)#(eU)#(eG)#(eU)#(eG)#(eA)#(eC)#(eU)#(eU)#(eG)#(eA)#(eU)#(eG)#(eA)#(eC)#(eA)#(eU)#(eC)#(eG)#(eG)
17	W-2174	(eU)#(eA)#(eU)#(eG)#(eU)#(eG)#(eA)#(eC)#(eU)#(eU)#(eG)#(eA)#(eU)#(eG)#(eA)#(eC)#(eA)#(eU)#(eC)#(eG)
18	W-2175	(eA)#(eU)#(eA)#(eU)#(eG)#(eU)#(eG)#(eA)#(eC)#(eU)#(eU)#(eG)#(eA)#(eU)#(eG)#(eA)#(eC)#(eA)#(eU)#(eC)
19	W-2176	(eA)#(eA)#(eU)#(eA)#(eU)#(eG)#(eU)#(eG)#(eA)#(eC)#(eU)#(eU)#(eG)#(eA)#(eU)#(eG)#(eA)#(eC)#(eA)#(eU)
20	W-2177	(eC)#(eA)#(eA)#(eU)#(eA)#(eU)#(eG)#(eU)#(eG)#(eA)#(eC)#(eU)#(eU)#(eG)#(eA)#(eU)#(eG)#(eA)#(eC)#(eA)
21	W-2178	(eG)#(eC)#(eA)#(eA)#(eU)#(eA)#(eU)#(eG)#(eU)#(eG)#(eA)#(eC)#(eU)#(eU)#(eG)#(eA)#(eU)#(eG)#(eA)#(eC)
22	W-2179	(eU)#(eG)#(eC)#(eA)#(eA)#(eU)#(eA)#(eU)#(eG)#(eU)#(eG)#(eA)#(eC)#(eU)#(eU)#(eG)#(eA)#(eU)#(eG)#(eA)
23	W-892	(eC)#(eC)#(eU)#(eC)#(eC)#(eA)#(eA)#(eU)#(eC)#(eC)#(eC)#(eU)#(eG)#(eC)#(eU)#(eU)#(eG)#(eG)#(eU)#(eG)
24	W-893	(eA)#(eC)#(eC)#(eU)#(eC)#(eC)#(eA)#(eA)#(eU)#(eC)#(eC)#(eC)#(eU)#(eG)#(eC)#(eU)#(eU)#(eG)#(eG)#(eU)
25	W-894	(eU)#(eA)#(eC)#(eC)#(eU)#(eC)#(eC)#(eA)#(eA)#(eU)#(eC)#(eC)#(eC)#(eU)#(eG)#(eC)#(eU)#(eU)#(eG)#(eG)
26	W-895	(eU)#(eU)#(eA)#(eC)#(eC)#(eU)#(eC)#(eC)#(eA)#(eA)#(eU)#(eC)#(eC)#(eC)#(eU)#(eG)#(eC)#(eU)#(eU)#(eG)
27	W-896	(eC)#(eU)#(eU)#(eA)#(eC)#(eC)#(eU)#(eC)#(eC)#(eA)#(eA)#(eU)#(eC)#(eC)#(eC)#(eU)#(eG)#(eC)#(eU)#(eU)
28	W-897	(eA)#(eC)#(eU)#(eU)#(eA)#(eC)#(eC)#(eU)#(eC)#(eC)#(eA)#(eA)#(eU)#(eC)#(eC)#(eC)#(eU)#(eG)#(eC)#(eU)
29	W-898	(eC)#(eA)#(eC)#(eU)#(eU)#(eA)#(eC)#(eC)#(eU)#(eC)#(eC)#(eA)#(eA)#(eU)#(eC)#(eC)#(eC)#(eU)#(eG)#(eC)
30	W-899	(eC)#(eC)#(eA)#(eC)#(eU)#(eU)#(eA)#(eC)#(eC)#(eU)#(eC)#(eC)#(eA)#(eA)#(eU)#(eC)#(eC)#(eC)#(eU)#(eG)
31	W-900	(eA)#(eC)#(eC)#(eA)#(eC)#(eU)#(eU)#(eA)#(eC)#(eC)#(eU)#(eC)#(eC)#(eA)#(eA)#(eU)#(eC)#(eC)#(eC)#(eU)
32	W-901	(eU)#(eA)#(eC)#(eC)#(eA)#(eC)#(eU)#(eU)#(eA)#(eC)#(eC)#(eU)#(eC)#(eC)#(eA)#(eA)#(eU)#(eC)#(eC)#(eC)
33	W-902	(eC)#(eU)#(eA)#(eC)#(eC)#(eA)#(eC)#(eU)#(eU)#(eA)#(eC)#(eC)#(eU)#(eC)#(eC)#(eA)#(eA)#(eU)#(eC)#(eC)
34	W-903	(eC)#(eC)#(eU)#(eA)#(eC)#(eC)#(eA)#(eC)#(eU)#(eU)#(eA)#(eC)#(eC)#(eU)#(eC)#(eC)#(eA)#(eA)#(eU)#(eC)
35	W-904	(eU)#(eC)#(eC)#(eA)#(eA)#(eU)#(eC)#(eC)#(eC)#(eU)#(eG)#(eC)#(eU)#(eU)#(eG)#(eG)#(eU)#(eG)#(eG)#(eG)
36	W-905	(eC)#(eC)#(eA)#(eA)#(eU)#(eC)#(eC)#(eC)#(eU)#(eG)#(eC)#(eU)#(eU)#(eG)#(eG)#(eU)#(eG)#(eG)#(eG)#(eA)
37	W-906	(eC)#(eA)#(eA)#(eU)#(eC)#(eC)#(eC)#(eU)#(eG)#(eC)#(eU)#(eU)#(eG)#(eG)#(eU)#(eG)#(eG)#(eG)#(eA)#(eG)
38	W-907	(eA)#(eA)#(eU)#(eC)#(eC)#(eC)#(eU)#(eG)#(eC)#(eU)#(eU)#(eG)#(eG)#(eU)#(eG)#(eG)#(eG)#(eA)#(eG)#(eC)
39	W-908	(eA)#(eU)#(eC)#(eC)#(eC)#(eU)#(eG)#(eC)#(eU)#(eU)#(eG)#(eG)#(eU)#(eG)#(eG)#(eG)#(eA)#(eG)#(eC)#(eU)
40	W-909	(eU)#(eC)#(eC)#(eC)#(eU)#(eG)#(eC)#(eU)#(eU)#(eG)#(eG)#(eU)#(eG)#(eG)#(eG)#(eA)#(eG)#(eC)#(eU)#(eC)
41	W-910	(eC)#(eC)#(eC)#(eU)#(eG)#(eC)#(eU)#(eU)#(eG)#(eG)#(eU)#(eG)#(eG)#(eG)#(eA)#(eG)#(eC)#(eU)#(eC)#(eA)
42	W-911	(eC)#(eC)#(eU)#(eG)#(eC)#(eU)#(eU)#(eG)#(eG)#(eU)#(eG)#(eG)#(eG)#(eA)#(eG)#(eC)#(eU)#(eC)#(eA)#(eU)
43	W-912	(eC)#(eU)#(eG)#(eC)#(eU)#(eU)#(eG)#(eG)#(eU)#(eG)#(eG)#(eG)#(eA)#(eG)#(eC)#(eU)#(eC)#(eA)#(eU)#(eC)
44	W-913	(eU)#(eG)#(eC)#(eU)#(eU)#(eG)#(eG)#(eU)#(eG)#(eG)#(eG)#(eA)#(eG)#(eC)#(eU)#(eC)#(eA)#(eU)#(eC)#(eA)
45	W-914	(eG)#(eC)#(eU)#(eU)#(eG)#(eG)#(eU)#(eG)#(eG)#(eG)#(eA)#(eG)#(eC)#(eU)#(eC)#(eA)#(eU)#(eC)#(eA)#(eC)
46	W-915	(eC)#(eU)#(eU)#(eG)#(eG)#(eU)#(eG)#(eG)#(eG)#(eA)#(eG)#(eC)#(eU)#(eC)#(eA)#(eU)#(eC)#(eA)#(eC)#(eU)
Chol-ASO	Chol-ASO	TegChol-(mG)#(mA)#(mG)#(rC)(rT)(rC)(rC)(rC)(rA)(rC)(rC)(rA)(rA)(rG)(rC)(rA)(rG)(rG)(rG)(rA)(rT)(rT)#(mG)#(mG)#(mA)
$\text{PMO}^{\text{PE44.1}}$	$\text{PMO}^{\text{PE44.1}}$	(pT)*(pC)*(pC)*(pA)*(pA)*(pT)*(pC)*(pC)*(pC)*(pT)*(pG)*(pC)*(pT)*(pT)*(pG)*(pG)*(pT)*(pG)*(pG)*(pG)*(pA)*(pG)*(pC)*(pT)*(pC)

Legend
2'-MOE A: (eA)
2'-MOE C: (eC)
2'-MOE G: (eG)
2'MOE U: (eU)
2'-Ome A: (mA)
2'-Ome C: (mC)
2'-Ome G: (mG)
2'Ome U: (mU)
RNA A: (rA)
RNA C: (rC)
RNA G: (rG)
RNA U: (rU)
Morpholine Ring A: (pA)
Morpholine Ring C: (pC)
Morpholine Ring G: (pG)
Morpholine Ring T: (pT)
Phosphorothioate bond :)#(
Phosphorodiamidate bond: *
