# Supplementary material for: Peptide-phosphorodiamidate morpholino oligomer therapy for dysferlinopathy induces pseudoexon skipping and restoration of functional protein
Source: JCI Insight. 2026 Jul 14;11(16):e204742. doi: 10.1172/jci.insight.204742 (PMC13502175; doi:10.1172/jci.insight.204742)

Unedited Western Blots

Dysferlin (Green)

Beta Actin (Red)

Figure 1D

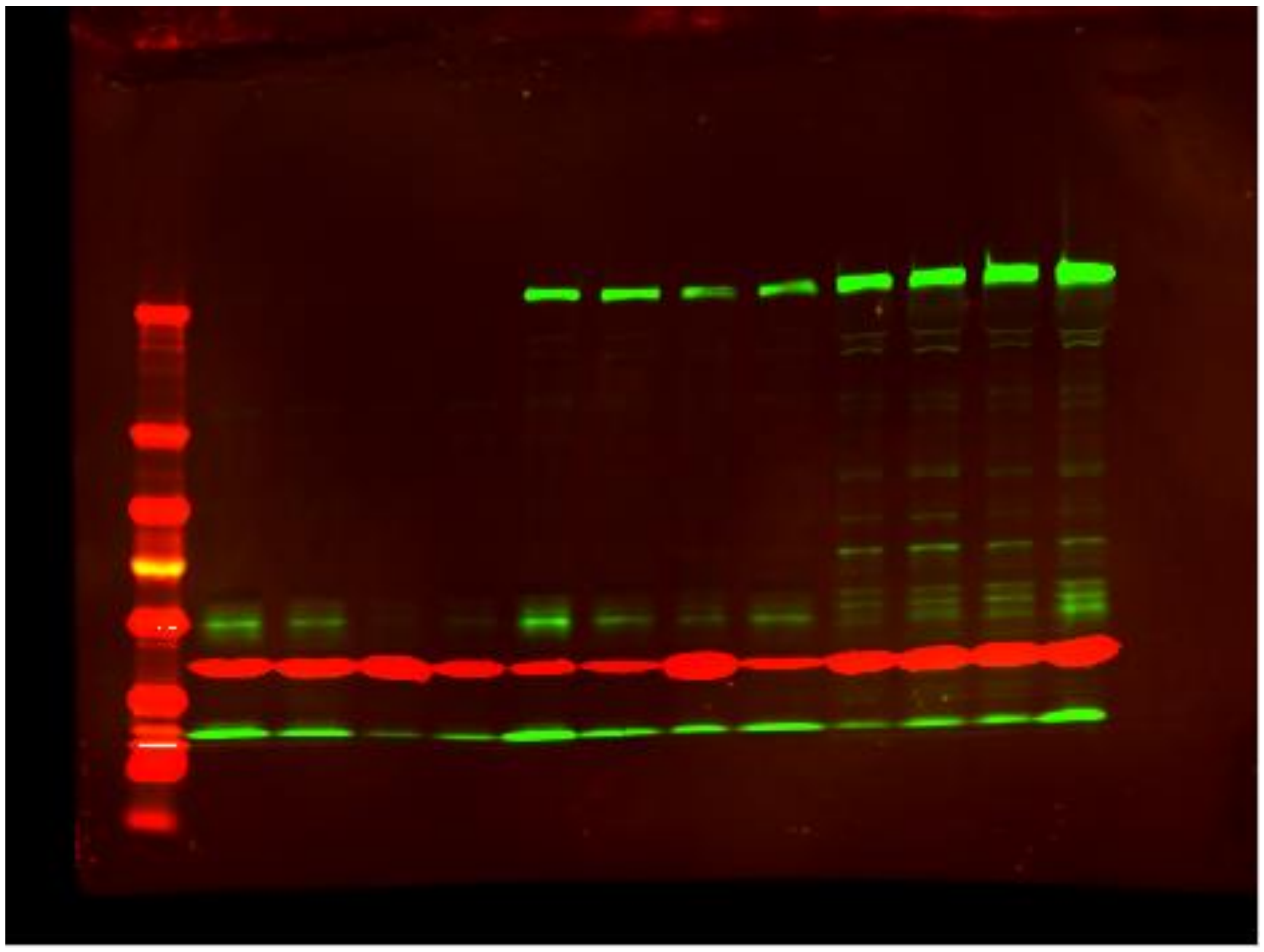

Figure 4C:

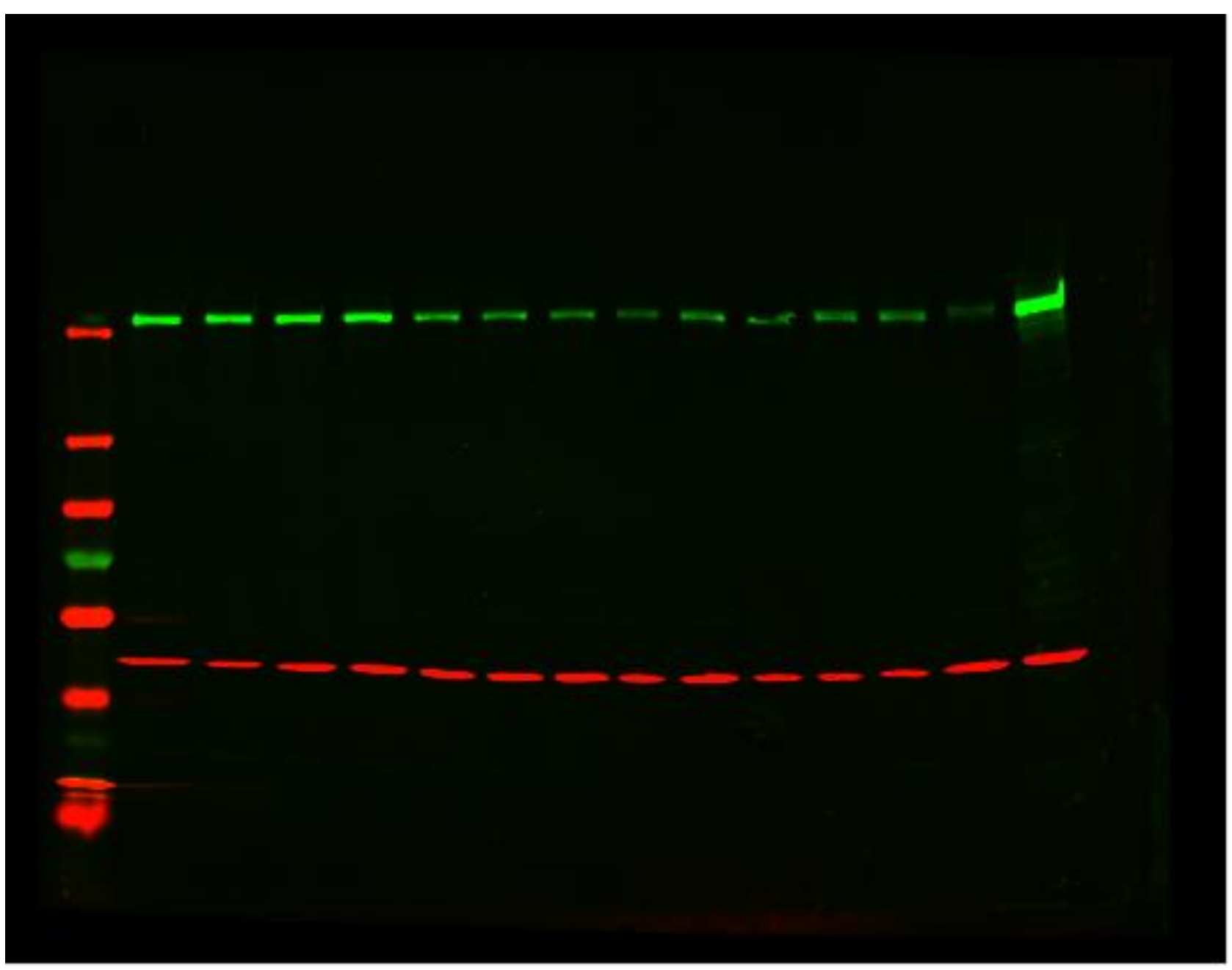

Figure 5G

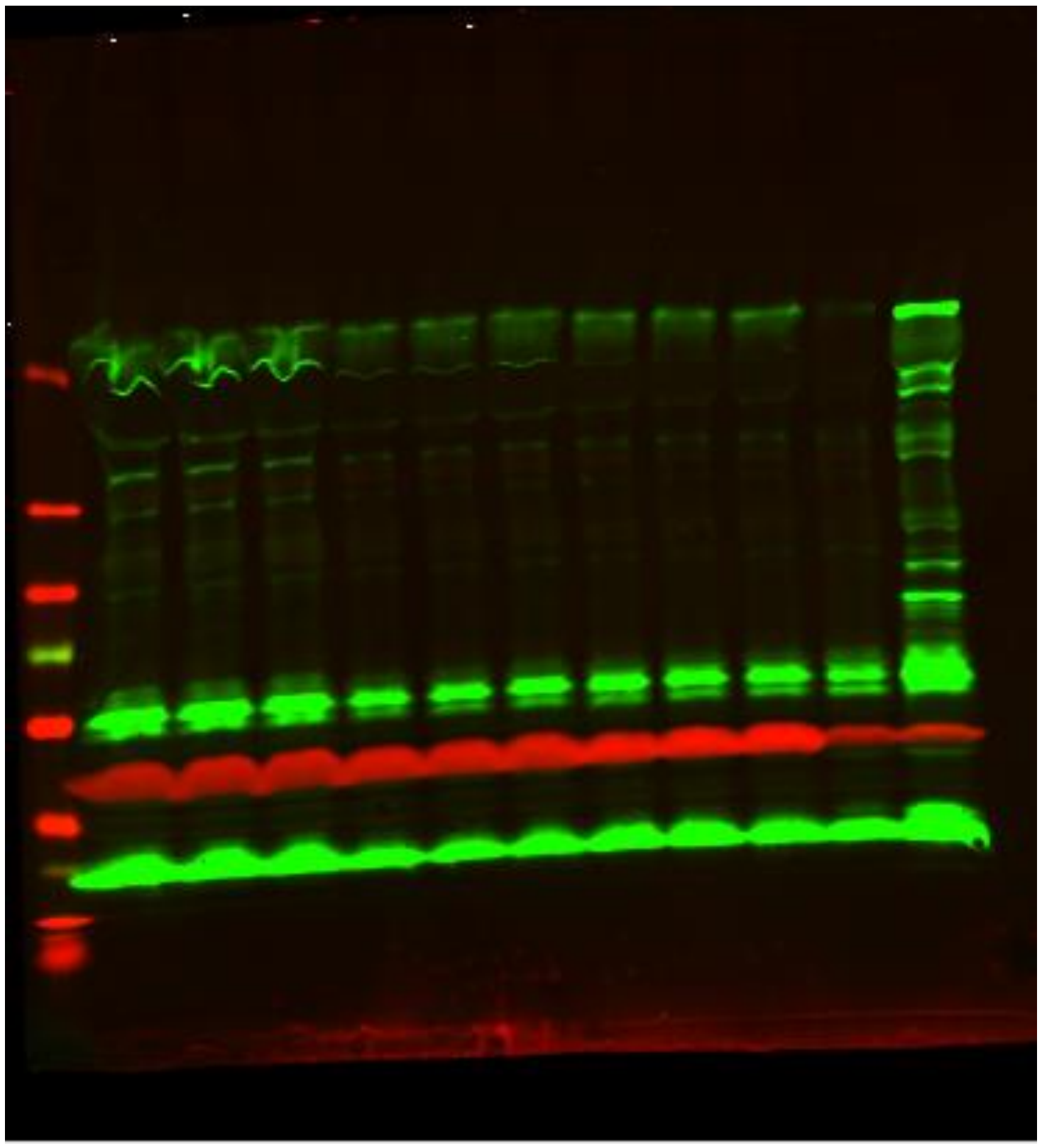

Figure 5H

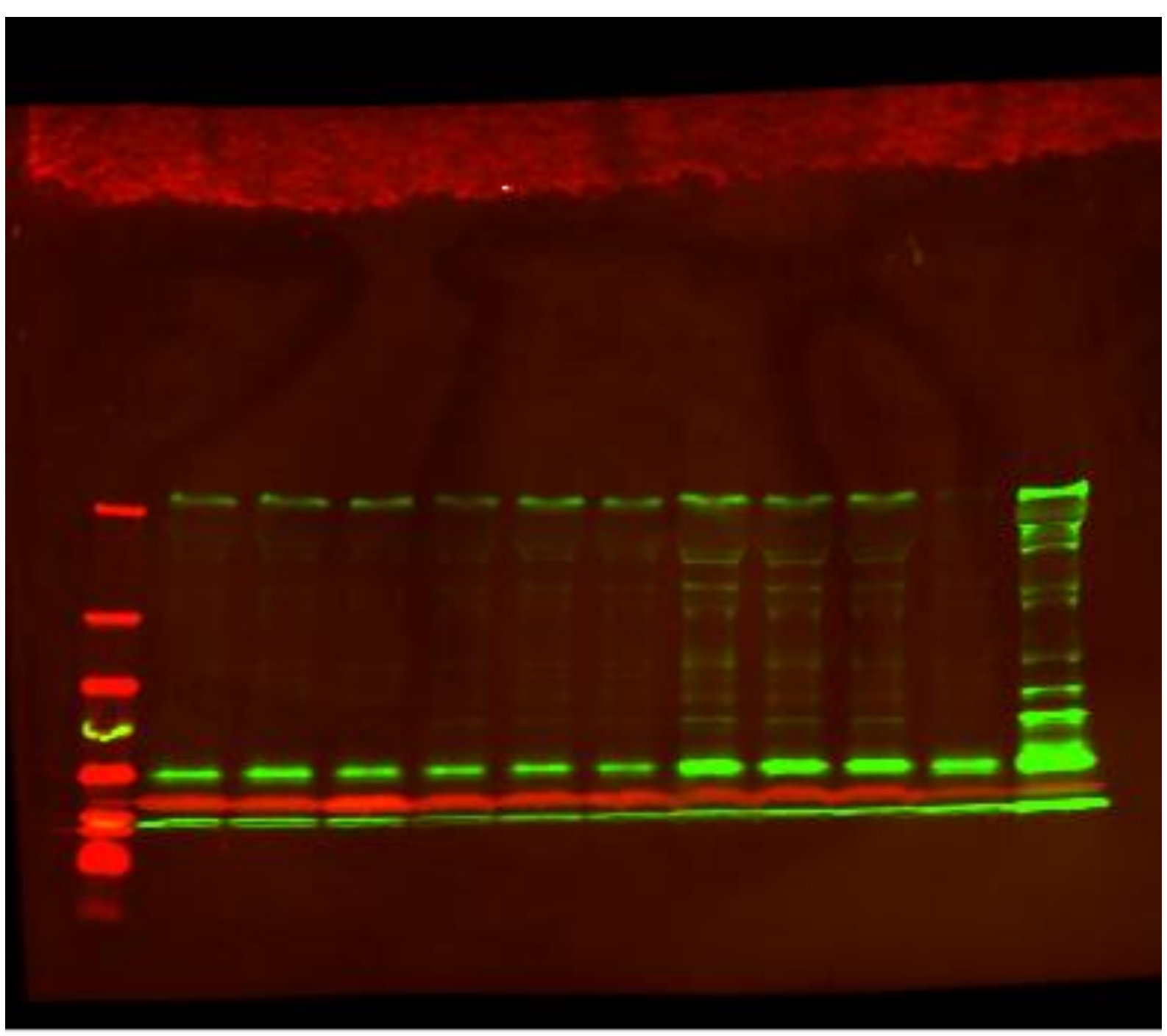

Figure 5I

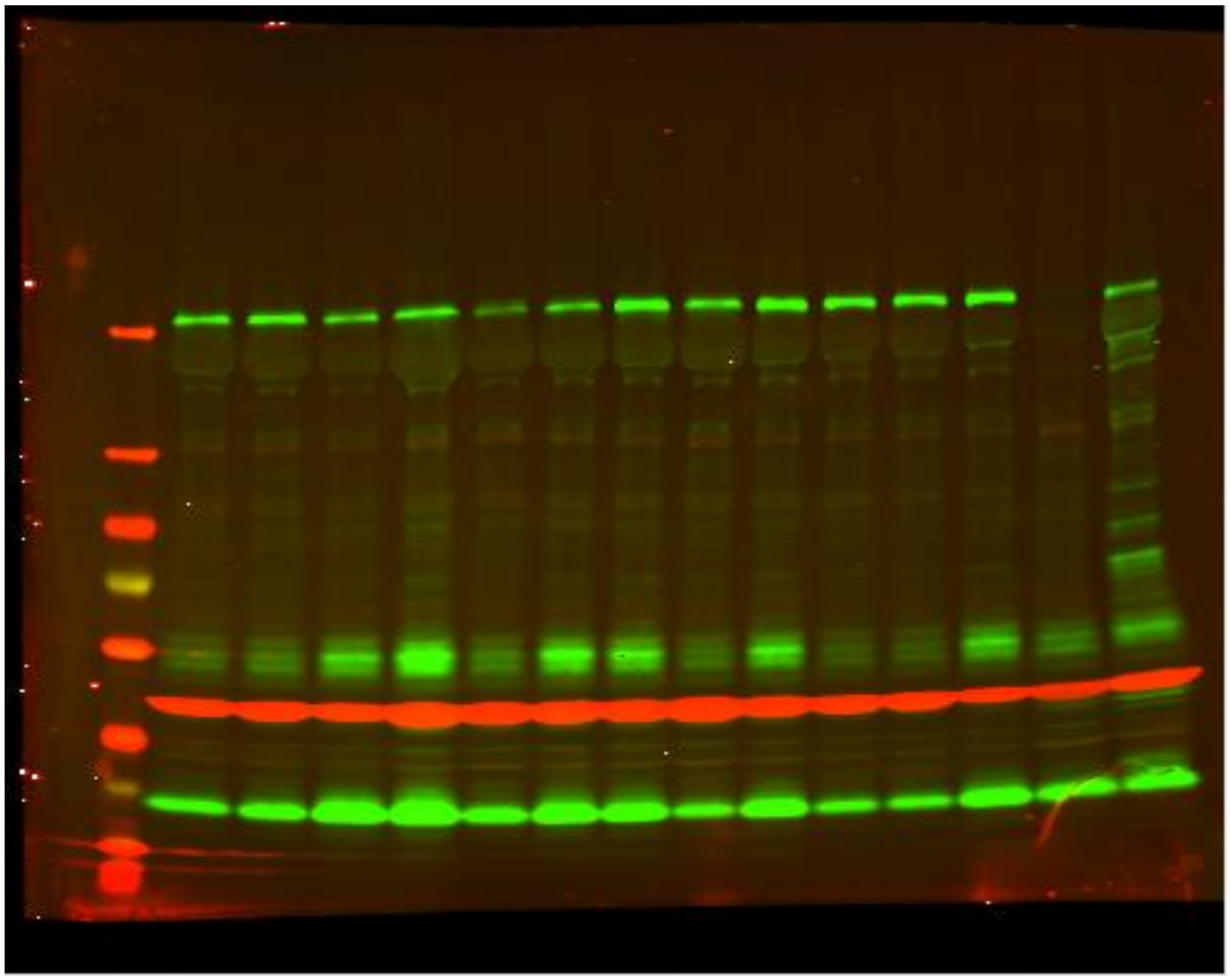

Supplemental Figure S8

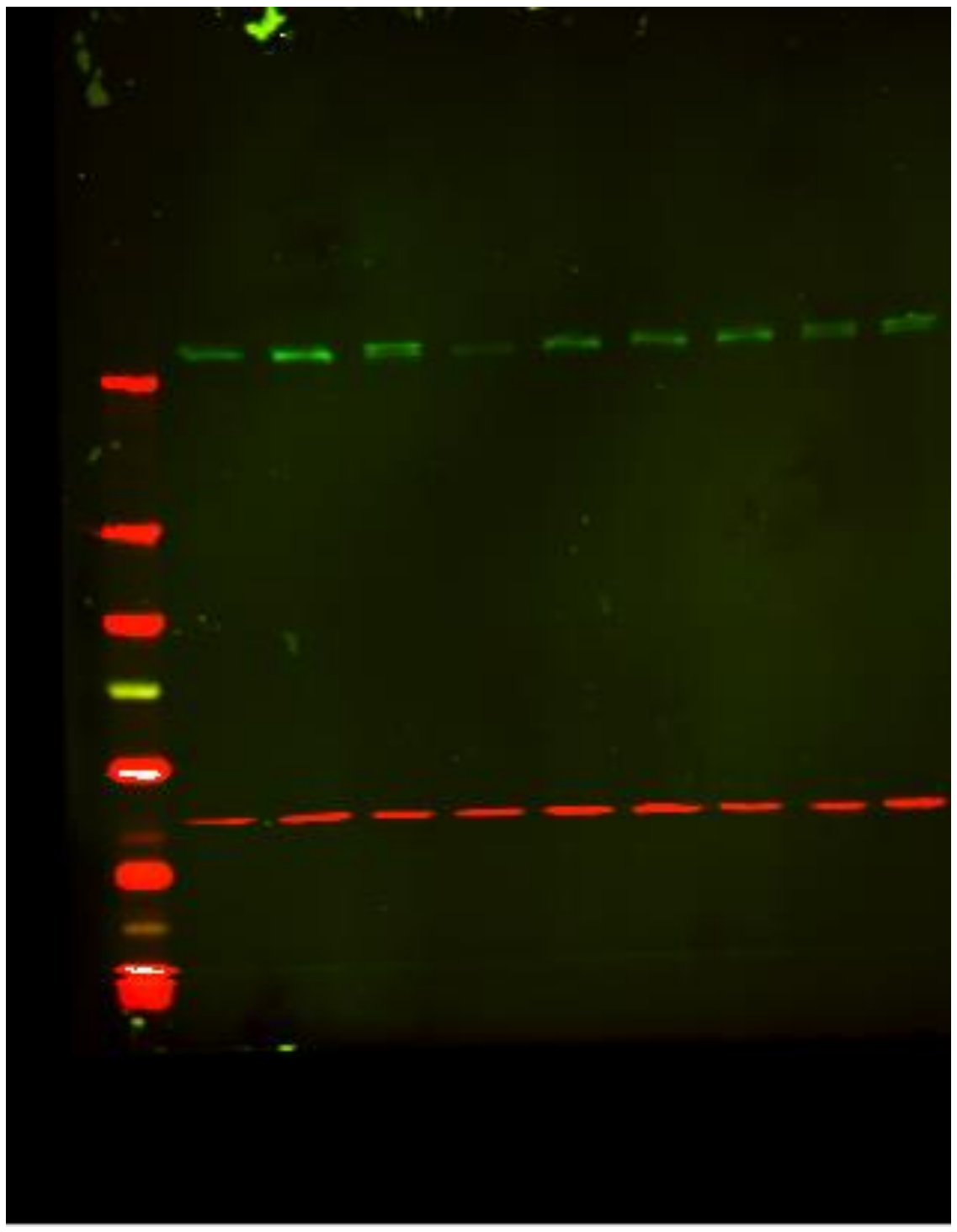

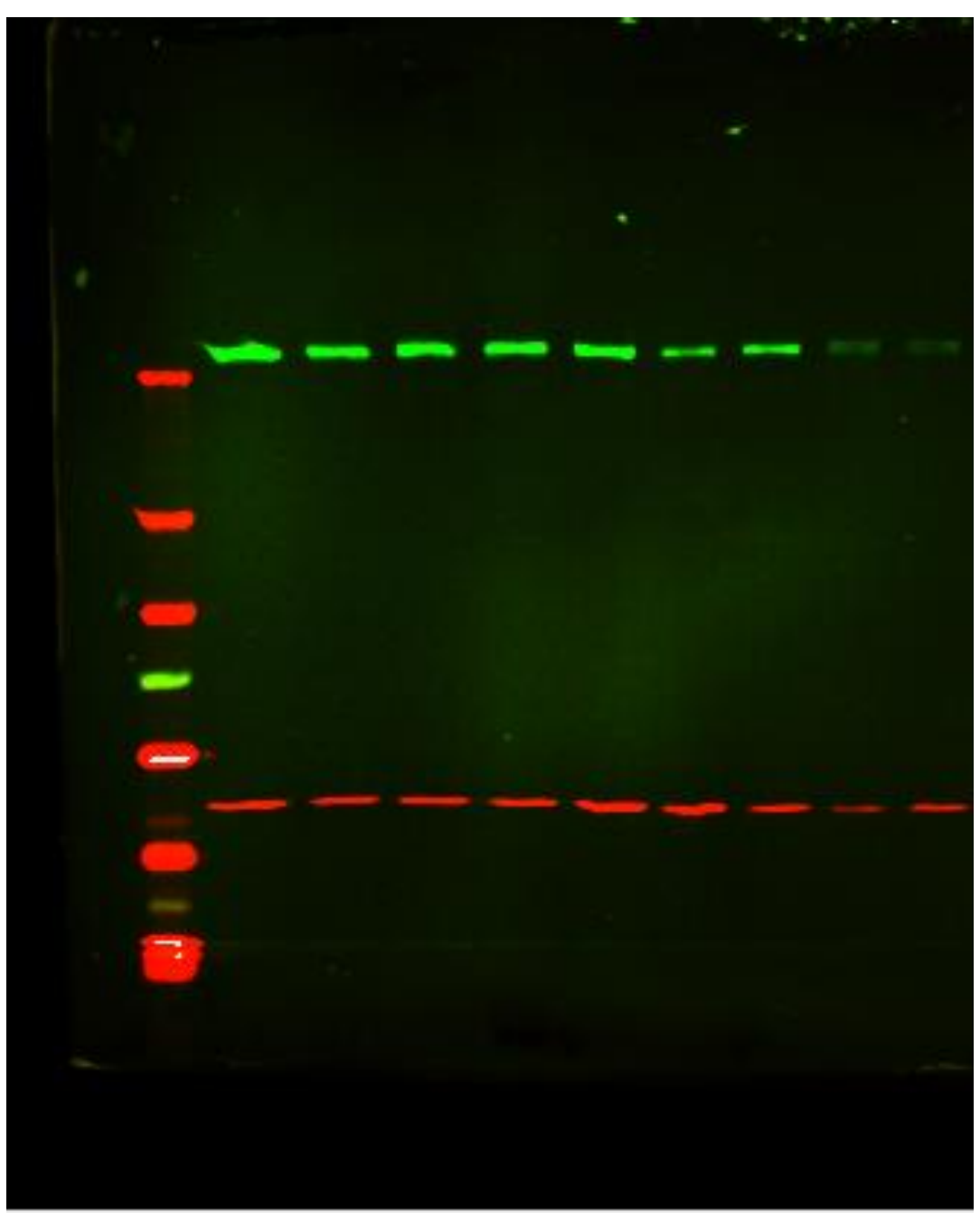

Supplement: Supplementary file 2 [file jciinsight-11-204742-s075.pdf]
